# CGRP‐Loaded ROS‐Responsive Hydrogel Restores Neuro‐Angiogenic Signaling to Promote Bone Regeneration in Diabetes‐Associated Periodontitis

**DOI:** 10.1002/advs.202506438

**Published:** 2025-08-04

**Authors:** Chaoning Zhan, Qingyi Dai, Jianhan Ren, Lijian Jin, Weiping Wang, Zhou Ye, James Kit Hon Tsoi, Yifan Lin

**Affiliations:** ^1^ Division of Paediatric Dentistry and Orthodontics Faculty of Dentistry The University of Hong Kong Hong Kong SAR China; ^2^ Division of Periodontology & Implant Dentistry Faculty of Dentistry The University of Hong Kong Hong Kong SAR China; ^3^ State Key Laboratory of Pharmaceutical Biotechnology The University of Hong Kong Hong Kong SAR China; ^4^ Department of Pharmacology & Pharmacy Li Ka Shing Faculty of Medicine The University of Hong Kong Hong Kong SAR China; ^5^ Laboratory of Molecular Engineering and Nanomedicine Dr. Li Dak‐Sum Research Centre The University of Hong Kong Hong Kong SAR China; ^6^ Applied Oral Sciences and Community Dental Care Faculty of Dentistry The University of Hong Kong Hong Kong SAR China

**Keywords:** bone regeneration, calcitonin gene‐related peptide, diabetes‐related periodontitis, ROS‐responsive hydrogel, sensory nerve, type H vessels

## Abstract

Diabetes exacerbates the development and progression of periodontitis through the aggravation of persistent inflammation and tissue destruction. While the impact of diabetes on peripheral sensory nerves is well‐documented, little is known about the role of diabetic neuropathy in bone destruction in diabetes‐associated periodontitis. Herein, a significant loss of periodontal nerves is observed in the diabetic state of db/db mice, with trigeminal ganglion neurons showing decreased autophagy. These mice exhibit decreased density of calcitonin gene‐related peptide (CGRP)^+^ nerves, correlating with the progression of diabetes and inflammatory state. Furthermore, diabetic mice with periodontitis show greater alveolar bone loss, which can be phenocopied by periodontal denervation. Importantly, CGRP receptor‐related components are found to be expressed in periodontal endothelial cells. In both diabetic and denervated periodontium, the loss of CGRP signaling is associated with the reduction of type H vessel density and coupled osterix^+^ osteoprogenitors. To elaborate further, an injectable reactive oxygen species‐responsive poly(vinyl alcohol) (PVA)/tsPBA hydrogel is developed for sustained CGRP delivery. Notably, the CGRP‐loaded hydrogels promote alveolar bone regeneration via inducing type H vessel formation in diabetic mice. The findings highlight that diabetes‐induced sensory nerve damage may exacerbate periodontitis‐induced bone loss, and CGRP@PVA/tsPBA hydrogels offer a promising therapeutic strategy for bone regeneration.

## Graphical abstract

This study shows that diabetes damages sensory nerve fibers, especially CGRP‐positive ones, in the periodontium and disrupts autophagy in trigeminal ganglion neurons, affecting bone homeostasis by inhibiting type H vessel formation. To address this, CGRP@PVA/tsPBA hydrogels are developed to release CGRP in response to ROS, which binds to endothelial cell receptors, promoting type H vessel formation and bone regeneration.

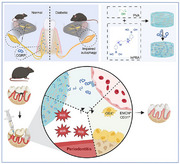

## 1 Introduction

Diabetes mellitus, a systemic disorder, adversely affects the onset and progression of periodontitis.^[^ ^1^ ^]^ Even after periodontal inflammation is controlled, tissue repair and regeneration are often compromised in patients with diabetes‐associated periodontitis.^[^ ^2^ ^]^ High blood glucose levels or hyperglycemia occur when there is insufficient insulin or insulin resistance in the human body. Hyperglycemia is frequently associated with microvascular dysfunction, immune dysregulation, and cellular senescence of stem cells.^[^ ^3^ ^]^ Furthermore, periodontitis can be aggravated by hyperglycemia‐induced reactive oxygen species (ROS) overproduction.^[^ ^4^ ^]^ Although the mechanisms behind impaired bone healing in diabetes‐associated periodontitis are gradually being uncovered, much remains poorly understood.

Peripheral neuropathy is another common complication of diabetes that typically leads to abnormalities in the structure and function of peripheral sensory nerves, beginning distally in the lower extremities.^[^ ^5^ ^]^ It is commonly characterized by the inability to sense the location, orientation, position, and movement of the body parts.^[^ ^5^ ^]^ Additionally, it reduces temperature and pain perception, leading to paresthesias and dysesthesias accompanied by neuropathic pain.^[^ ^6^ ^]^ Since sensory nerve fibers not only serve a sensory function but also play a critical regulatory role by releasing neuropeptides,^[^ ^7^ ^]^ diabetes has been proven to delay tissue repair via affecting sensory nerves from the dorsal root ganglia.^[^ ^7^, ^8^ ^]^ However, the contribution of sensory nerves originating from the trigeminal ganglion to alveolar bone homeostasis in diabetes‐associated periodontitis remains unaddressed.

The trigeminal system supplies sensory innervation to the stomatognathic systems. Over the last decade, advances in trigeminal system research have revealed significant diabetes‐induced pathological changes, including the loss of nerve fibers within the buccal mucosa and chronic abnormal somatic sensations.^[^ ^9^ ^]^ Although trigeminal ganglia also innervate periodontium, the alterations in the density and modulatory function of sensory nerves in the diabetic periodontium remain to be elucidated. The periodontal tissues are innervated by sensory nerves that express calcitonin gene‐related peptide (CGRP) and substance P (SP).^[^ ^10^ ^]^ As sensory neuropeptides, CGRP and SP can be secreted by sensory nerve endings into the ambient microenvironment to regulate periodontal metabolism. CGRP acts as a positive protagonist of bone metabolism and tissue healing. For example, CGRP is abundant in perivascular nerves and can enhance osteogenesis by increasing angiogenesis.^[^ ^11^ ^]^ Additionally, CGRP restores bone marrow mesenchymal stem cell activity and osteogenic differentiation in the presence of inflammation.^[^ ^12^ ^]^ Furthermore, this neuropeptide creates an ideal microenvironment to promote tissue healing by inducing the pro‐repair phenotype of macrophages.^[^ ^7^, ^13^ ^]^ The receptor of SP is also expressed in several kinds of cells, including osteoblasts, osteoclasts, fibroblasts, macrophages, etc.^[^ ^14^ ^]^ The effects of SP on tissue healing may be mainly achieved by inducing an acute neuroinflammatory response, which enables the progression to the reparative phase.^[^ ^15^ ^]^ Subsequently, SP decreases the time span of the inflammation phase and activates M2 macrophage polarization.^[^ ^16^ ^]^ Collectively, these findings show that sensory neuropeptides serve as critical modulators in inflammation and tissue repair. Therefore, the potential damage to periodontal innervation probably leads to more severe inflammation and impaired tissue regeneration in diabetes‐associated periodontitis.

This study aimed to investigate the role of sensory nerves in periodontal bone homeostasis within the context of diabetes‐associated periodontitis. Subsequently, we constructed an engineered niche by incorporating relevant neuropeptides (CGRP) into a ROS‐responsive hydrogel to enhance bone regeneration in diabetes‐associated periodontitis.

## 2 Results

### 2.1 Hyperglycemia‐Induced Neural Changes in Periodontium

**Figure 1:**
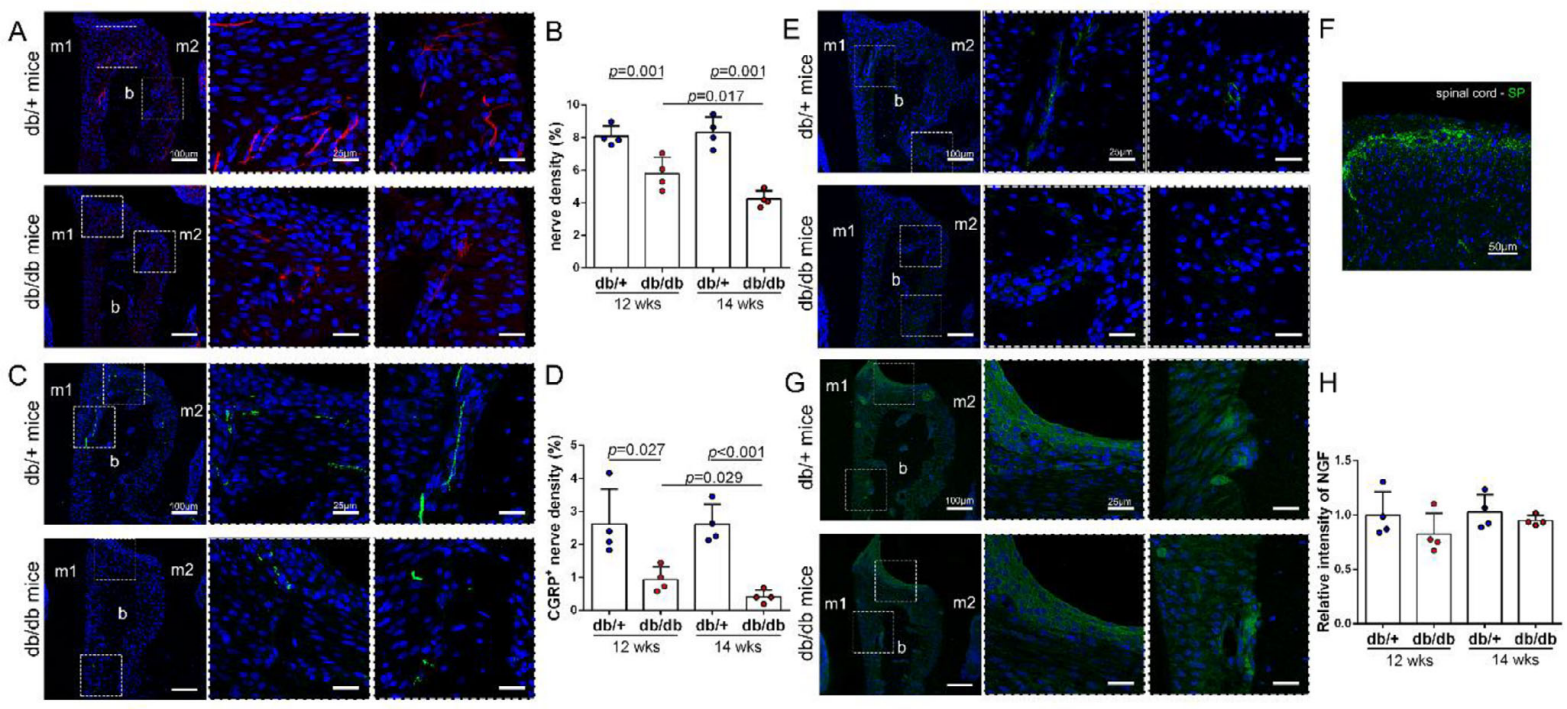
Neural changes in the periodontium of db/db mice. A) Immunofluorescence staining showing the change in the density of β 3 Tubulin^+^ nerves (red). B) Quantitative analysis of β 3 Tubulin^+^ nerve density confirming the less density in periodontal tissues of db/db mice compared to db/+ mice (n = 4). C) Immunofluorescence images of CGRP showing the change in periodontal CGRP^+^ nerves (green). D) Quantification showing a reduction in the periodontal CGRP^+^ nerves in db/db mice (n = 4). E) SP‐immunoreactive nerves (green) in the periodontium of db/+ mice and db/db mice. F) SP staining obtained from positive samples verifying the validity of staining. G) There was no change in NGF expression (green) in the periodontium between the two groups. H) Quantitative analysis of NGF staining confirming that the periodontal NGF expression in db/db mice is indistinguishable from that of db/+ mice (n = 4). b, bone; m1, the first molars; m2, the second molars; CGRP, calcitonin gene‐related peptide; NGF, nerve growth factor; SP, substance P.

The body weight of db/db mice was twofold higher compared to db/+ mice (Figure S1A,B, Supporting Information). And the glucose level in db/db mice was ≈2.5‐fold higher compared with db/+ mice (Figure S1C, Supporting Information). There were no clear histological differences between the periodontium of 14‐week‐old db/+ mice and db/db mice (Figure S2, Supporting Information). Then, periodontium was analyzed for the expression of peripheral neural marker β 3 tubulin. However, β 3 tubulin‐labeled nerve density was obviously decreased within the epithelial and periodontal ligament areas of db/db mice (**Figure** **1**A), and the results indicated that density was lower at a later time point (14 weeks) than that at an earlier time point (12 weeks, Figure 1B; 28.5 and 49.2% reduction at weeks 12 and 14, respectively). Corresponding to β 3 tubulin^+^ nerve fibers, CGRP^+^ nerve fibers showed the same decreasing pattern of change, which, after quantification, exhibited a 64.1 and 83.9% reduction at weeks 12 and 14, respectively (Figure 1C,D). The density of SP^+^ nerve fibers was also assessed by immunostaining. However, only single nerve fibers were labeled with SP (Figure 1E), and any issues with the staining analysis itself have been excluded by a positive control (Figure 1F). Since nerve growth factor (NGF) affects the sprouting of sensory nerves and its expression correlates with diabetes in some tissues, we decided to assess the expression of NGF in the periodontium.^[^ ^17^ ^]^ Whereas the quantification of NGF fluorescence intensity did not demonstrate significant differences between the two groups (Figure 1G,H). Taken together, these results indicate that although NGF expression is not influenced by diabetes, the neural microenvironment is impaired by this systemic disease.

### 2.2 Hyperglycemia‐Induced Neural Changes in Trigeminal Ganglia

**Figure 2:**
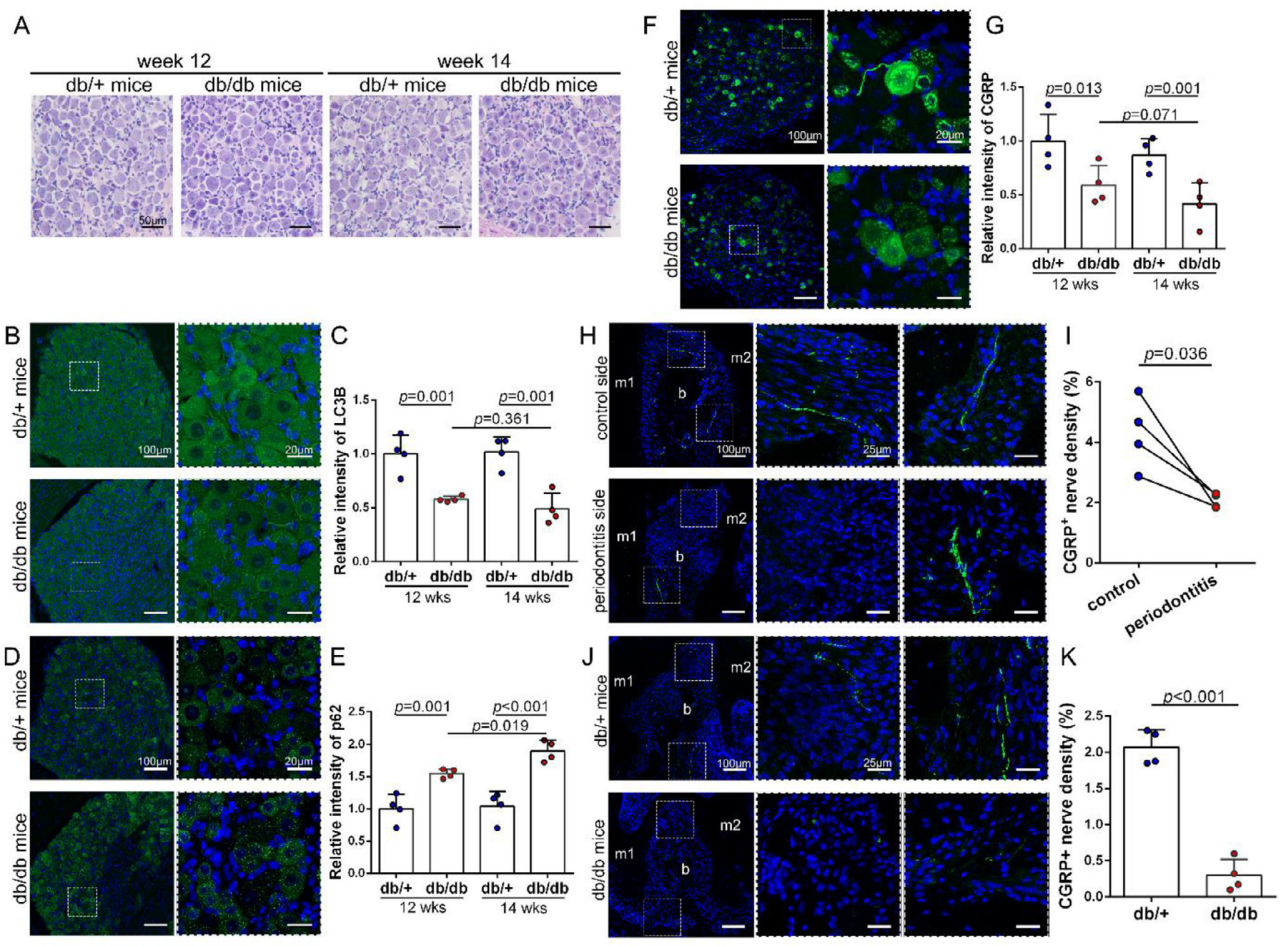
Neural changes in the trigeminal ganglia of db/db mice. A) Images of H&E staining in the trigeminal ganglia reveal no histological variation between db/+ and db/db mice. B) The LC3B expression (green) was lower in the trigeminal ganglia of db/db mice compared to control mice. C) Quantitative analysis of LC3B staining in different groups (n = 4). D) The p62 protein (green) was expressed at a higher level in the trigeminal ganglia from db/db mice. E) Quantitative evaluation of p62 expression levels. F) The expression of CGRP (green) was lower in the trigeminal ganglia of db/db mice compared to control mice. G) Quantitative analysis of CGRP expression in ganglia (n = 4). H and I) Representative images and quantitative analysis demonstrating that the density of CGRP^+^ nerves (green) was lower on the periodontitis sides compared to control sides in db/+ mice. (n = 4). J and K) Representative images and quantitative analysis demonstrating that the CGRP ^+^ nerves (green) were less dense in periodontitis tissues in db/db mice compared to db/+ mice (n = 4). b, bone; m1, the first molars; m2, the second molars; CGRP, calcitonin gene‐related peptide; LC3B, microtubule‐associated protein 1 light chain 3 beta.

We next turned to explore changes in the trigeminal ganglion as a result of diabetes and whether the peripheral sensory nerve changes were due to the gangalionic abnormalities. Whereas, as shown in **Figure** **2**A, H&E staining showed that there was no appreciable difference between the two groups. In addition, no apoptotic cells were detected by TUNEL staining (Figure S3, Supporting Information). As another fundamental process in the cell, autophagy maintains cellular homeostasis through degrading proteins and organelles when cells are exposed to stress.^[^ ^18^ ^]^ To assess whether peripheral neuropathy is associated with dysregulated autophagy in diabetic trigeminal ganglion neurons, we evaluated autophagy using microtubule‐associated protein 1 light chain 3 beta (LC3B) and p62 staining in trigeminal ganglion tissues. Immunofluorescence (IF) analysis revealed a significant downregulation of LC3B expression in db/db mice compared with controls, reflecting a 42.0 and 52.8% reduction in staining intensity at weeks 12 and 14 (Figure 2B,C). In contrast, a 54.6 and 85.9% increase of p62 expression at weeks 12 and 14 was observed (Figure 2D,E). Meanwhile, we evaluated the trigeminal ganglionic CGRP protein content and found that there was less CGRP in the trigeminal ganglia of db/db mice compared with db/+ mice (Figure 2F,G). Due to the fact that dental pulp also receives a dense supply of nerve endings originating from the trigeminal ganglion,^[^ ^19^ ^]^ we also found a significant decrease in CGRP immunoreactive nerves in the dental pulp of db/db mice (Figure S4, Supporting Information).

In addition to the diabetic periodontium, a lower density of CGRP^+^ nerves in the gingival epithelium and periodontal ligament was also found in the inflammatory periodontium compared with the contralateral healthy periodontium in db/+ mice (Figure 2H,I). In the inflammatory periodontium from db/db mice, there was a significantly decreasing density of CGRP^+^ nerves in the periodontal ligament under the root furcation of the second molars compared with the inflammatory periodontium from db/+ mice (up to 85.5%) (Figure 2J,K). This result means that diabetes and inflammation have a synergistic effect on decreasing CGRP^+^ nerve density.

### 2.3 Sensory Denervation Aggravates Bone Resorption in Wild‐Type Mice Showing Similar Phenotypes to db/db Mice

**Figure 3:**
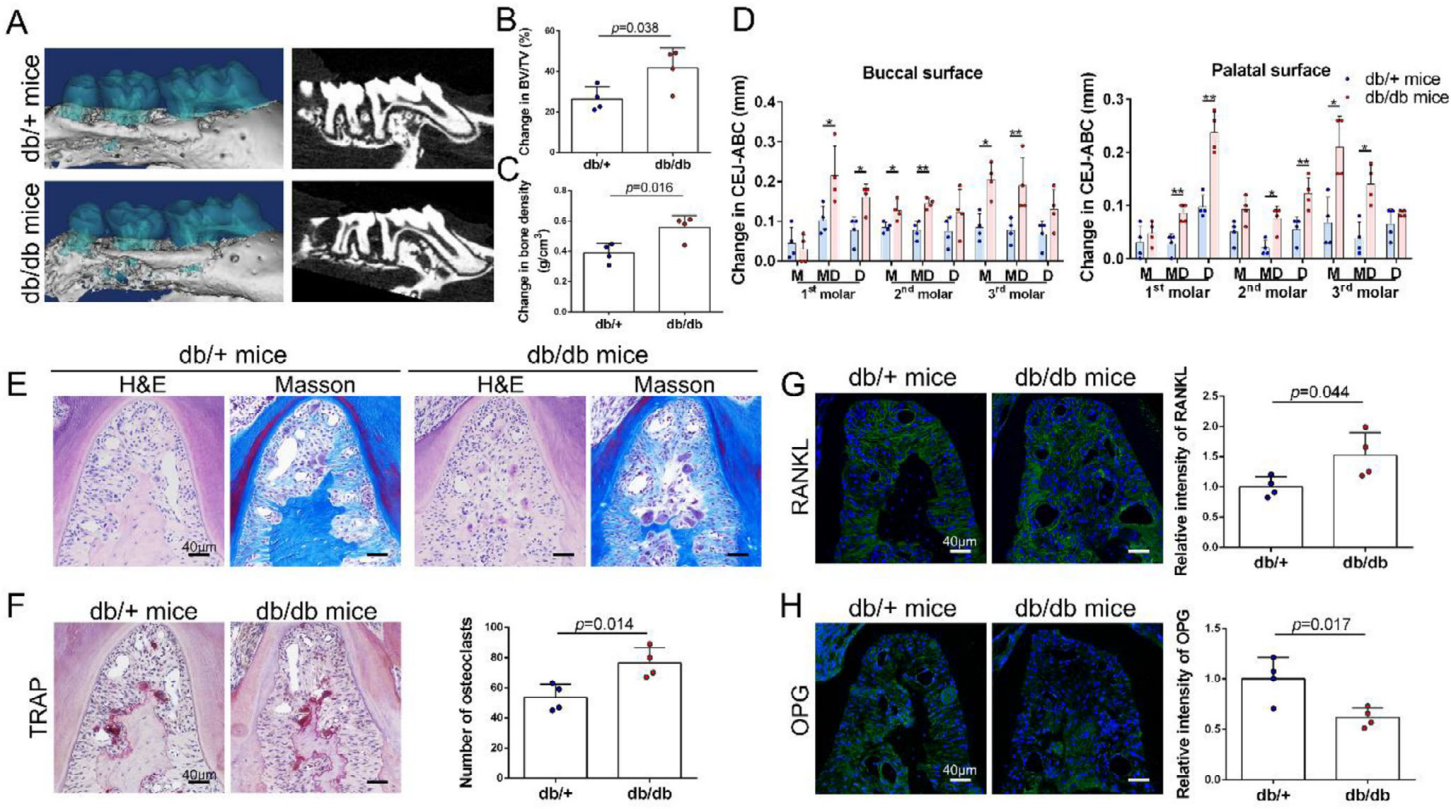
Ligature induces more severe alveolar bone loss in db/db mice compared to db/+ mice. A) 3D representation showing the aggravation of alveolar bone loss in db/db mice with periodontitis. B and C) Change in bone volume/tissue volume and bone density within the root furcation area of the second molar (n = 4). D) Change in cementoenamel junctions to the alveolar bone crest (n = 4). E) H&E and Masson's trichrome of periodontitis tissue showing more severe alveolar bone resorption in db/db mice. F) TRAP staining revealing more osteoclasts in periodontitis tissue in db/db mice (n = 4). G) RANKL immunofluorescence staining (green) of periodontitis tissue in db/+ mice and db/db mice (n = 4). H) OPG immunofluorescence staining (green) of periodontitis tissue in db/+ mice and db/db mice (n = 4). OPG, osteoprotegerin; RANKL, receptor activator of nuclear factor kappa‐B ligand. ^*^ *P* < 0.05; ^**^ *P* < 0.01.

To determine the influence of diabetes on periodontitis, silk sutures were tied on the upper‐second molars of db/+ and db/db mice. Given that it has been reported that diabetes itself could lead to alveolar bone loss without ligature placement or the introduction of bacteria,^[^ ^20^ ^]^ changes in the index of bone resorption were calculated by subtracting its value from that of the corresponding contralateral site. According to micro‐CT analysis, the changes in bone volume/tissue volume (BV/TV) and bone density in the furcation area of the second molar with periodontitis were significantly higher in db/db mice (**Figure** **3**A–C). Besides, the change of the cementoenamel junction to the alveolar bone crest (CEJ‐ABC) distances was also higher in db/db mice than in db/+ mice (Figure 3D). The histological sections from db/db mice showed more prominent bone resorption, with an increasing number of TRAP‐positive osteoclasts (Figure 3E,F). The IF staining showed a higher expression of receptor activator of NF‐κB ligand (RANKL) and a lower expression of osteoprotegerin (OPG) in db/db mice compared with db/+ mice (Figure 3G,H).

**Figure 4:**
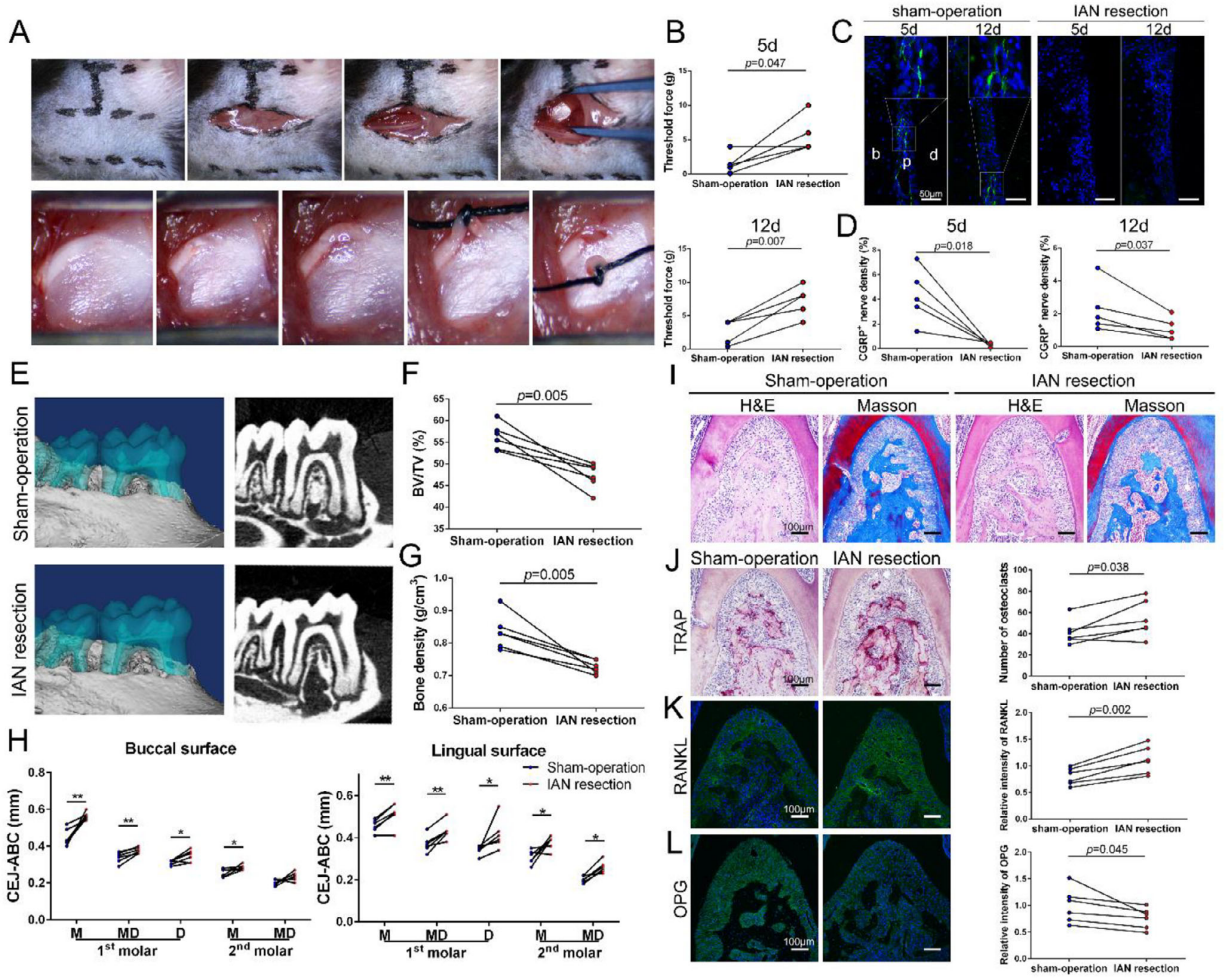
IAN resection phenocopies aggravated alveolar bone resorption in db/db mice with periodontitis. A) Photographs of the experimental periodontal sensory denervation operation, showing the procedure to expose the IAN. Finally, the IAN was ligated and lifted using a silk suture, and one millimeter of the IAN was dissected away. B) Von Frey tests at the lower lip (n = 5). C) Histological analysis of CGRP^+^ nerves (green) at 5 and 12 days postoperatively. D) Quantification of CGRP^+^ nerve density on days 5 and 12 after IAN resection (n = 5). E) 3D representation of periodontitis in IAN‐denervated and sham‐operated sides. F and G) Bone volume/tissue volume and bone density within the root furcation area of the first molar (n = 6). H) Cementoenamel junctions to the alveolar bone crest (n = 6). I and J) H&E, Masson's trichrome, and TRAP staining of periodontitis tissue in the IAN‐denervated and sham‐operational sides (n = 6). K and L) RANKL and OPG immunofluorescence staining (green) of periodontitis tissue in the IAN‐denervated and sham‐operational sides (n = 6). b, bone; p, periodontal ligament; d, dentin. IAN, inferior alveolar nerve; CGRP, calcitonin gene‐related peptide; RANKL, receptor activator of NF‐κB ligand; OPG, osteoprotegerin. ^*^ *p* < 0.05; ^**^ *p* < 0.01.

To mimic the effects of diabetes on periodontal sensory neural inactivity, we performed inferior alveolar nerve (IAN) axotomy on the left side without damage to vessels (**Figure** **4**A). The periodontium with intact peripheral nerves on the right side was used as a control. After a five‐day recovery period, a ligature was placed around the mandibular first molar for seven days to induce periodontitis. Hence, we detected the mechanical touch threshold at the lower lips and the density of CGRP^+^ nerve endings in periodontal ligaments on days 5 and 12. The touch threshold increased significantly after axotomy (Figure 4B), and the CGRP^+^ nerve density reduced by 92.6 and 53.0% on days 5 and 12 (Figure 4C,D). The absence of sensory nerves resulted in more severe bone loss (Figure 4E), which was evident through decreased BV/TV and bone density compared to the sham‐operation sides (Figure 4F,G). Similarly, the CEJ‐ABC distances in denervated first molars were significantly higher than in contralateral innervated sides (Figure 4H). Histological staining further validated these observations, showing more severe bone resorption in the root furcation region in the denervated periodontium under inflammatory conditions (Figure 4I). TRAP staining revealed increased osteoclasts in IAN resection sides (Figure 4J). A higher RANKL and reduced OPG expression levels were observed through IF staining (Figure 4K,L).

### 2.4 Decreased Type H Vessel Formation in the Diabetic and Denervated Periodontium

**Figure 5:**
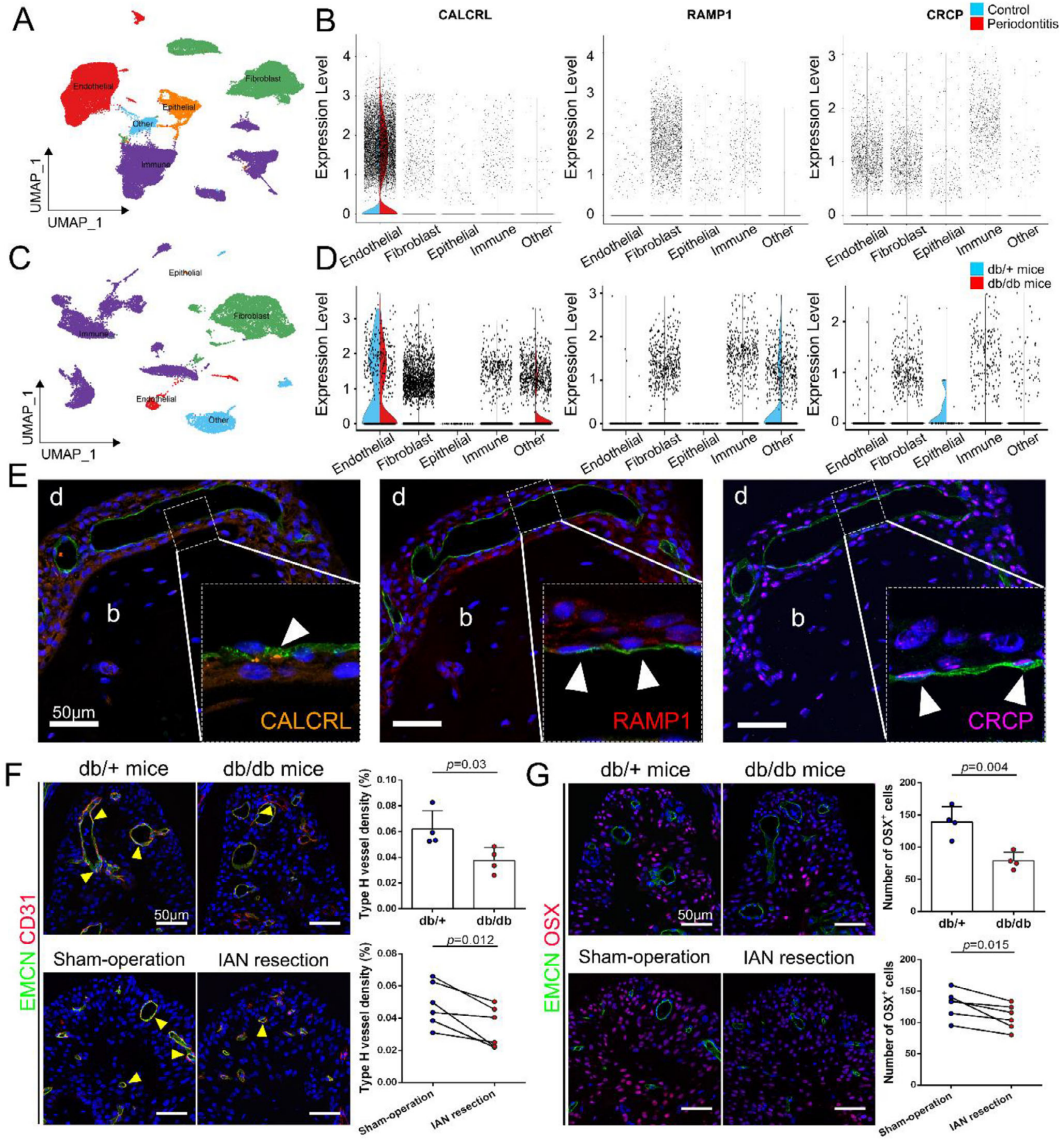
The expression of CGRP receptor‐related proteins in endothelial cells and the observation of downregulated type H vessels and couple osteoprogenitors in the periodontium of db/db mice or sensory denervated periodontium. A and B) Single‐cell type expression of CGRP receptors in human gingival tissues. C and D) Single‐cell type expression of CGRP receptors in mouse gingival tissues. E) Representative immunofluorescence images of EMCN co‐staining with CALCRL (orange), RAMP1 (red), and CRCP (violet). White triangles: endothelial cells expressing CGRP receptor‐related proteins. F) immunofluorescence staining of type H vessels, which highly express CD31 (red) and EMCN (green, n = 4 or 6). Yellow triangles: type H vessels. G) Representative immunofluorescence images of OSX^+^ cells (violet, n = 4 or 6). b, bone; d, dentin; CGRP, calcitonin gene‐related peptide; CALCRL, calcitonin receptor‐like receptor; CRCP, CGRP receptor component protein; EMCN, endomucin; OSX, Osterix; RAMP1, receptor activity‐modifying protein‐1.

The functional CGRP receptor is composed of three proteins: CGRP receptor component protein (CRCP), calcitonin receptor‐like receptor (CALCRL), and receptor activity‐modifying protein‐1 (RAMP1).^[^ ^21^ ^]^ To identify the main target cell types of the CGRP in the periodontium, a single‐cell‐type expression analysis of the three proteins was conducted based on the single‐cell RNA‐seq data for humans under normal or inflammatory conditions and mouse gingival tissues under healthy or diabetic conditions. Cell compartments were mainly classified into four types that are endothelial cells, fibroblasts, immune cells, and epithelial cells (**Figure** **5**A,C). Figure 5B showed that the CALCRL and CRCP were mainly enriched in human endothelial cells, while RAMP1 was enriched in human fibroblasts. Figure 5D demonstrated that CALCRL was also highly expressed in the endothelial cells of the mouse gingival tissues. Meanwhile, although RAMP1 and CRCP were not highly expressed in these endothelial cells, both genes were still expressed by endothelial cells (Figure 5D). To further confirm the expression of CGRP receptors in the endothelial cells within the periodontium, we performed IF staining on the mouse periodontal ligament and found that endomucin^+^ (EMCN^+^) endothelial cells expressed all three proteins (Figure 5E).

The vasculature, especially type H vessels, is actively involved in hard and soft tissue healing.^[^ ^22^ ^]^ To assess the impact of diabetes and sensory denervation on type H vessel formation, respectively, IF double staining of CD31 and EMCN was performed. In db/+ mice, type H endothelium density was 39.4% higher than in db/db mice (Figure 5F). The denervation surgery reproduced consistent results, i.e., the 29.4% reduction of type H vessel density in the sensory denervated periodontium (Figure 5F). Because endothelial cells displaying the phenotype of type H vessels were closely associated with osteogenesis by osterix^+^ (OSX^+^) skeletal progenitors,^[^ ^23^ ^]^ we also measured the number of OSX^+^ cells. Consistent with the results of type H vessels, the number of OSX^+^ cells showed a 43.5 and 16.0% decline in the periodontium of db/db mice and the sensory denervated periodontium (Figure 5G).

### 2.5 Fabrication and Characterization of ROS‐Sensitive Hydrogels

**Figure 6:**
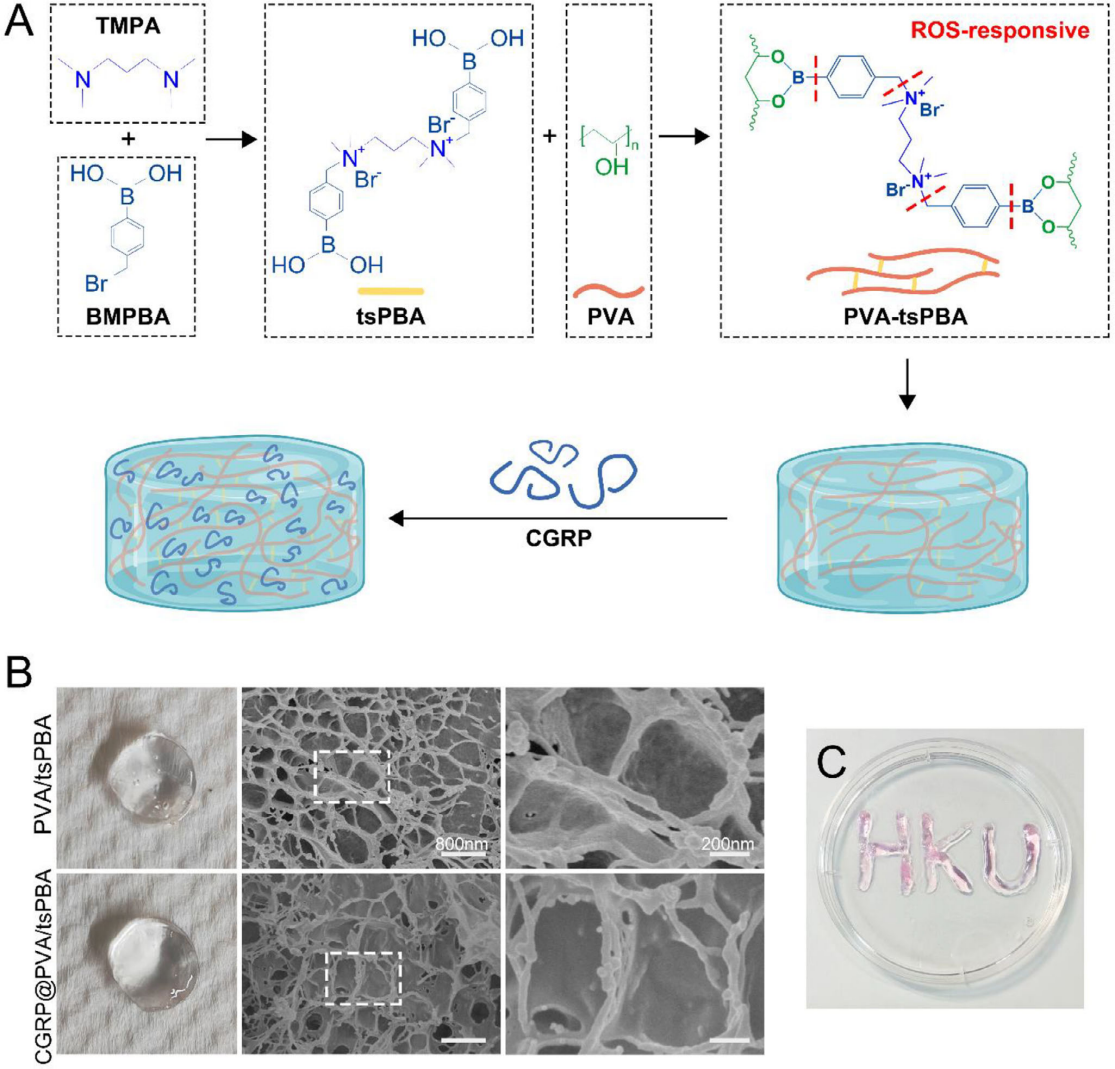
Preparation and characterization of CGRP@PVA/tsPBA hydrogels. A) Schematic diagram showing the design principle for ROS‐responsive hydrogels. B) Gross morphology and scanning electron microscopy of porous hydrogels, showing that the induction of CGRP into the hydrogel scaffolds did not affect the hydrogel structure. C) Photographs were taken of the letters “HKU”, which were formed by extruding PVA/tsPBA hydrogels, stained with dye, through a dual syringe. CGRP, calcitonin gene‐related peptide. PVA, poly(vinyl alcohol).

Under diabetic conditions, the excessive generation of ROS causes the imbalance of periodontal homeostasis.^[^ ^24^ ^]^ Additionally, ROS induces damage to blood vessels, leading to endothelial dysfunction.^[^ ^25^ ^]^ Introducing a ROS‐sensitive group into the hydrogel scaffold can make it responsive to ROS accumulation. Here, N^1^‐(4‐boronobenzyl)‐N^3^‐(4‐boronophenyl)‐N,^1^N^1^,N^3^,N^3^‐tetramethylpropane‐1,3‐diaminium (tsPBA) was used to cross‐link poly(vinyl alcohol) (PVA) by a dual syringe to obtain a ROS‐responsive hydrogel (**Figure** **6**A). The proton nuclear magnetic resonance spectrum of tsPBA linker matched its desired chemical structure well (Figure S5, Supporting Information), confirming the successful synthesis of the ROS‐responsive linker. The CGRP loaded into the hydrogel did not affect the hydrogel formation process (Figure 6B). The clear pore structure of the two hydrogels was revealed by scanning electron microscopy observation, and the addition of CGRP did not influence the porous structure of the hydrogels (Figure 6B). The injectability of PVA/tsPBA hydrogels was investigated by staining the hydrogels with a dye and writing “HKU” in Figure 6C.

**Figure 7:**
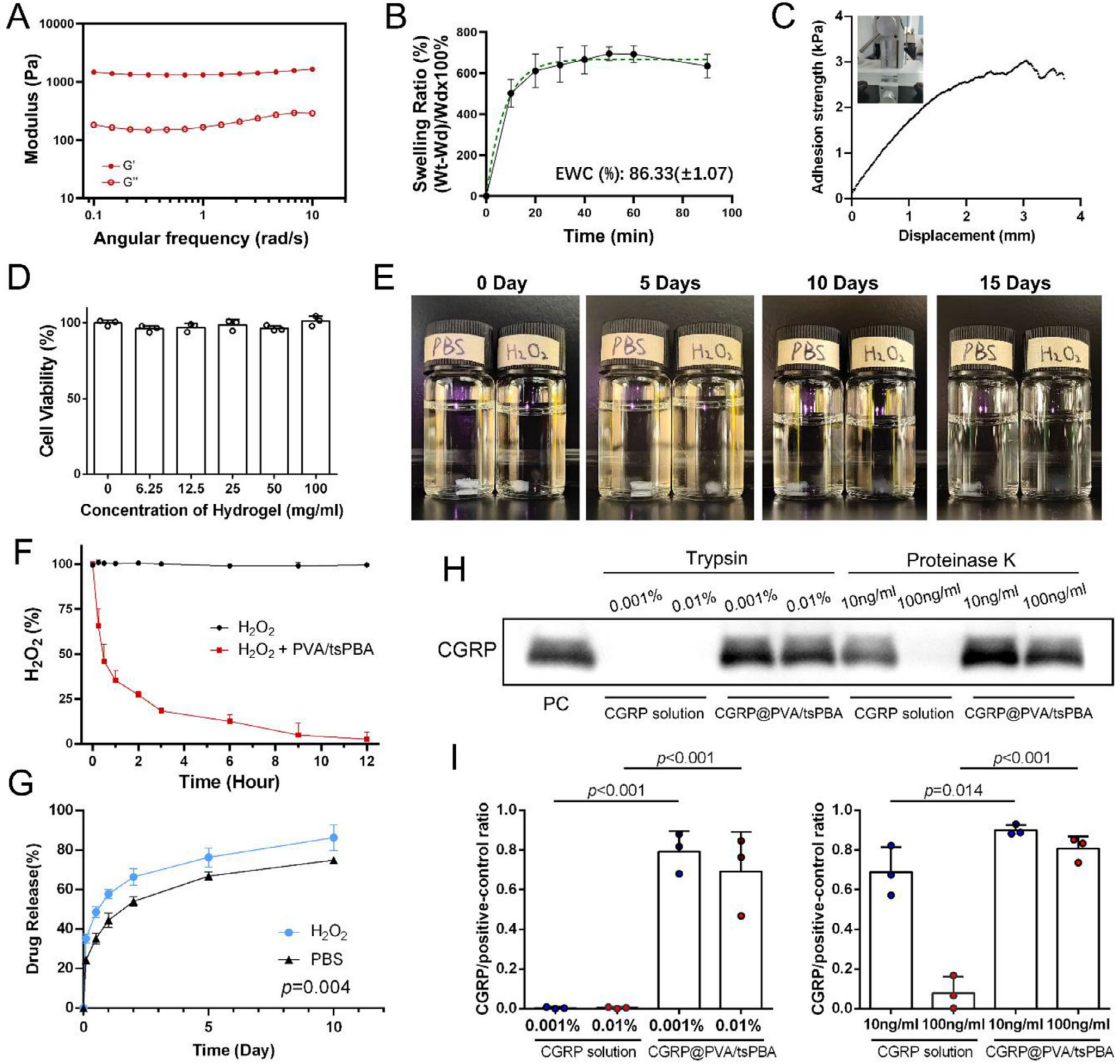
Properties of CGRP@PVA/tsPBA hydrogels. A) Rheological frequency analysis of the hydrogels' storage (G′) and loss (G″) moduli. B) Swelling ratio of PVA/tsPBA hydrogels (n = 3). C) The adhesive capacity of the PVA/tsPBA hydrogel on glass. D) The cell viability of HUVECs treated with different concentrations of hydrogels (n = 3). E) Morphological changes of PVA/tsPBA hydrogels in PBS with or without H_2_O_2_ addition (1 mM). F) The free radical‐scavenging ability of the hydrogels (n = 3). G) Release profiles of CGRP from hydrogels at 1 mM of H_2_O_2_ (n = 3, repeated measure ANOVA). H and I) Western blot analysis showing the amount of protected CGRP under the treatment with trypsin and proteinase K (n = 3). FITC, fluorescein isothiocyanate; CGRP, calcitonin gene‐related peptide; PVA, poly(vinyl alcohol).

In the rheological assessments, the storage modulus (G′) of the hydrogels exceeded the loss modulus (G″), suggesting the establishment of a flexible hydrogel as opposed to a viscous liquid (**Figure** **7**A). The water absorption abilities of the hydrogels were evaluated using equilibrium water content (EWC) and swelling kinetics tests. The hydrogels displayed EWC values above 80% and swelling ratios exceeding 600% (Figure 7B). The adhesive strength of hydrogels on glass was ≈3.01 kPa when subjected to a concentrated load at 3 mm displacement (Figure 7C). The CCK8 assay showed no cytotoxicity of endothelial cells, even at a high hydrogel concentration (up to 100 mg mL^−1^, Figure 7D). The presence of H_2_O_2_ accelerated the degradation of the hydrogel compared to a pure PBS solution, resulting in nearly complete degradation within 15 days (Figure 7E). Apart from the almost completely degraded hydrogel on day 15, scanning electron microscopy was used to observe hydrogels immersed in the H_2_O_2_ solution at three other time points. As shown in Figure S6 (Supporting Information), the hydrogel framework was gradually destroyed over time, further demonstrating its ROS responsiveness. Following incubation with the PVA/tsPBA hydrogel, 65% of H_2_O_2_ present in the solution was eliminated within the first hour. Furthermore, the hydrogels almost completely scavenged H_2_O_2_ within 12 h (Figure 7F). The release rate of CGRP from the hydrogel was enhanced after incubating with an H_2_O_2_ solution, attributable to the ROS‐responsive capacity of the hydrogel (Figure 7G). Additionally, the structural stability of the loaded CGRP in the hydrogels was evaluated. Western blotting results showed that CGRP encapsulated in the hydrogels maintained its structural properties and was protected from proteolytic degradation in contrast to unloaded CGRP (Figure 7H,I).

### 2.6 CGRP@PVA/tsPBA Hydrogels Promote Osteogenesis in Diabetic Periodontitis‐Induced Bone Loss

**Figure 8:**
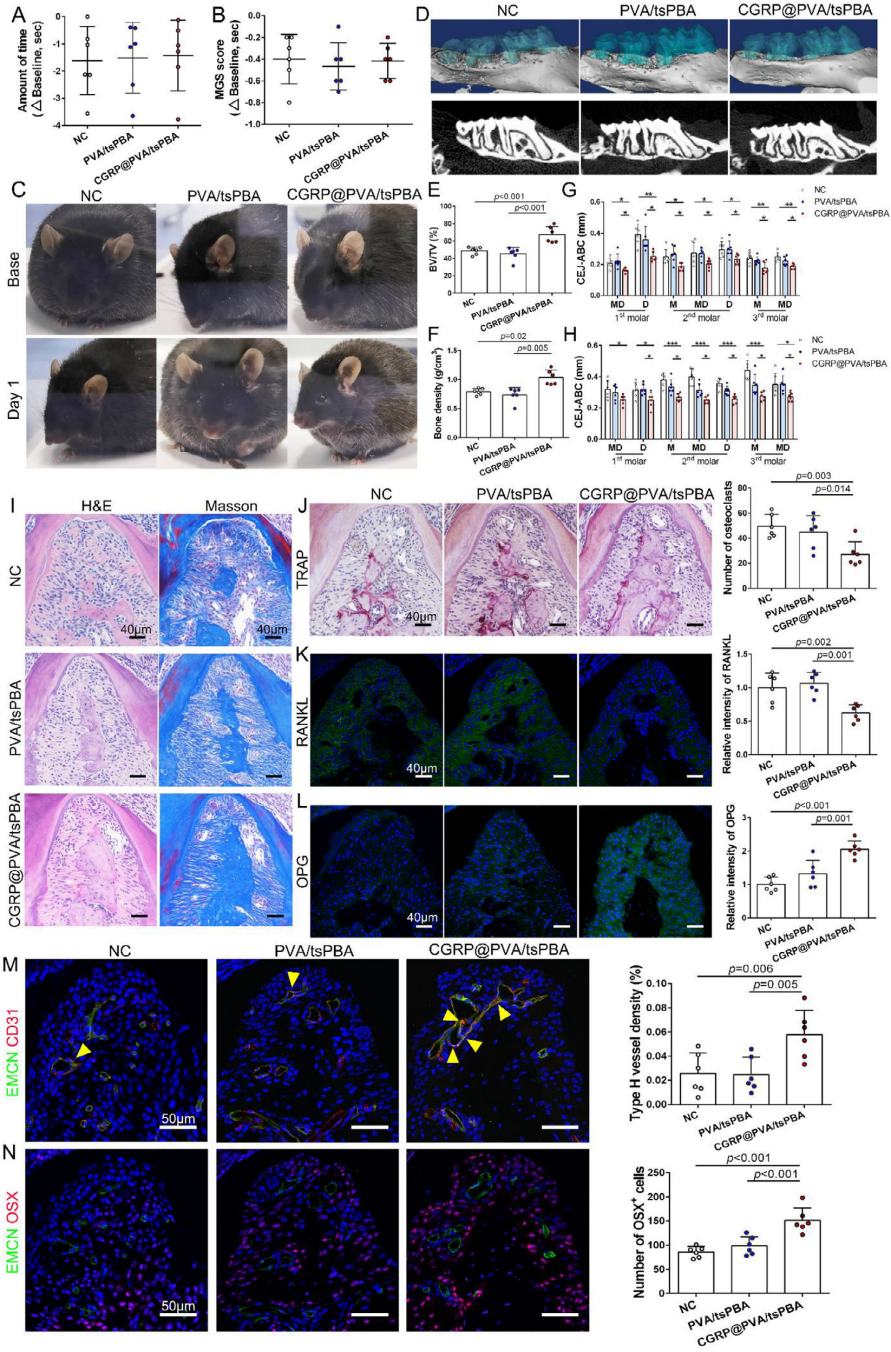
The effect of CGRP@PVA/tsPBA hydrogels on promoting bone regeneration in diabetes‐associated periodontitis‐induced bone loss. A) Quantification of the spontaneous face grooming response to different treatments, showing no pain inducement from the application of hydrogels (n = 6). B) Mean MGS scores of mice in the control group and db/db mice treated with PVA/tsPA hydrogels or PVA/tsPBA/CGRP hydrogels (n = 6). C) Representative images one day before and after the removal of ligatures and injection of hydrogels. D) Micro‐CT 3D reconstruction in control, PVA/tsPBA, and CGRP@PVA/tsPBA groups. E and F) Bone volume/tissue volume ratio and bone density within the root furcation area of the second molar (n = 6). G and H) Cementoenamel junctions to the alveolar bone crest on the buccal and palatal sides, respectively (n = 6). I) H&E and Masson's trichrome staining of a representative sagittal view of the 2D cross‐section of alveolar bone regeneration. J) Osteoblast staining by TRAP (n = 6). K and L) IF staining for RANKL (green) and OPG (green) in each group (n = 6). M and N) IF staining of type H vessels (yellow) and OSX^+^ osteoprogenitors (violet). Yellow triangles: type H vessels. CGRP, calcitonin gene‐related peptide; OPG, osteoprotegerin; PVA, poly(vinyl alcohol); RANKL, receptor activator of NF‐κB ligand. ^*^ *p* < 0.05; ^**^ *p* < 0.01; ^***^ *p* < 0.001.

Since CGRP participates in pain sensation in the peripheral nervous system, spontaneous pain‐like behaviors were monitored and measured after the application of CGRP‐loaded hydrogels. There was no significant difference in spontaneous face grooming and grimace scale scoring among the three groups (**Figure** **8**A–C). Hence, the introduction of CGRP into the periodontium does not induce pain behavior. Before investigating the therapeutic effect of CGRP@PVA/tsPBA on diabetes‐associated periodontitis‐induced bone loss, the IF staining of CGRP was performed, and the results showed that CGRP levels were significantly higher in the CGRP@PVA/tsPBA group compared to the two other groups (Figure S7, Supporting Information). Micro‐CT imaging clearly showed a significant alveolar bone formation in the CGRP@PVA/tsPBA group, while there were no obvious differences between the NC group and the PVA/tsPBA group (Figure 8D). Specifically, the BV/TV in the CGRP@PVA/tsPBA group was significantly higher than in the other two groups, with an average increase of 38.5 and 49% compared to the NC group and PVA/tsPBA group, respectively (Figure 8E). Additionally, as expected, bone density was 31.0 and 40.5% higher in the CGRP@PVA/tsPBA group than that in the other two groups (Figure 8F). The CEJ‐ABC distances were also shortest in the CGRP@PVA/tsPBA group (Figure 8G,H). Histologically, the H&E staining and Masson's trichrome staining demonstrated a higher quality of bone regeneration after treatment with CGRP‐loaded hydrogels (Figure 8I). Additionally, the number of TRAP^+^ osteoclasts was lowest in the same group (Figure 8J). Furthermore, the IF staining showed the lowest RANKL expression and the highest OPG expression in the PVA/tsPBA/CGRP group (Figure 8K,L). CGRP treatment significantly increased type H vessel formation and the number of OSX^+^ cells (Figure 8M,N). Finally, we reanalyzed the BV/TV ratio and bone density in the root furcation area on day 0 (when ligatures were removed) and day 28 (after ligature removal and hydrogel application). Both indices showed a significant increase by day 28 (Figure S8A,B, Supporting Information). Similarly, the number of OSX^+^ osteoprogenitors increased after 28 days of hydrogel application (Figure S8C, Supporting Information). Therefore, the greater bone formation observed in the CGRP@PVA/tsPBA group results from the regenerative effects of the hydrogels, and a higher number of osteoprogenitors was newly generated rather than merely maintained after hydrogel application.

## 3 Discussion

**Figure 9:**
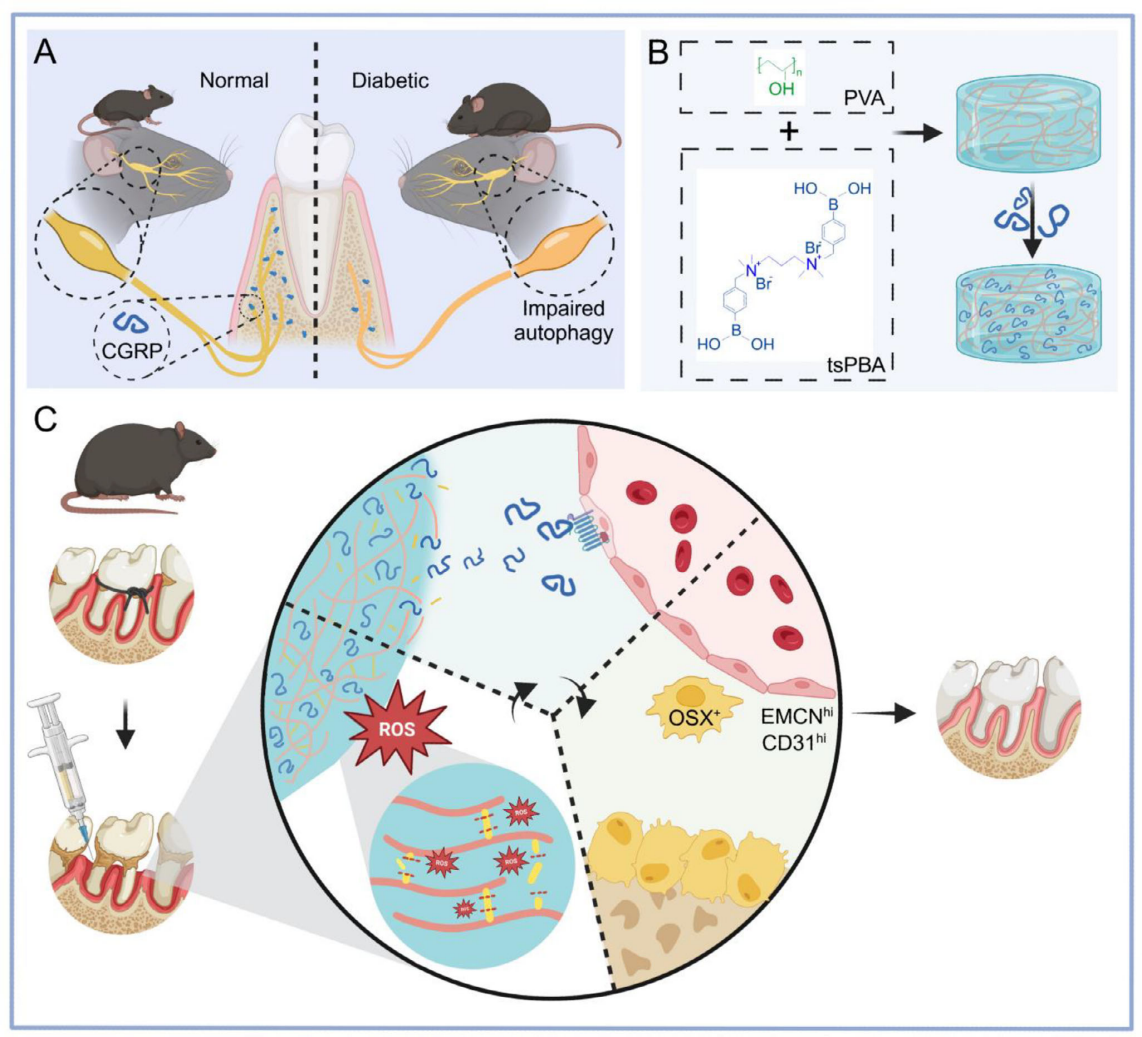
Schematic diagram of the impact of diabetes on periodontal innervation and the mechanism by which ROS‐responsive hydrogels encapsulating CGRP treat diabetes‐associated periodontitis. A) Schematic illustration showing the impaired sensory nerve fibers, particularly those with CGRP‐positive nerve endings, in diabetic periodontium, which results from dysregulated autophagy in trigeminal ganglion neurons. B) Outline of the process for preparing CGRP@PVA/tsPBA hydrogels. C) The administration of the hydrogels in cases of diabetes‐associated periodontitis can rectify the neural microenvironment by releasing CGRP in response to ROS stimulation. This released CGRP binds to receptors expressed by endothelial cells, promoting the formation of type H vessels and subsequently leading to alveolar bone formation.

There are several potential mechanisms by which diabetes could exacerbate periodontal inflammation and bone loss. In this study, we showed that diabetic sensory neuropathy directly affects periodontitis by harming the function of CGRP^+^ nerve fibers in periodontium, leading to impaired formation of type H vessels and OSX^+^ skeletal progenitors. Therefore, this mechanism could be one of the potential reasons for increasing bone resorption under diabetic conditions. Furthermore, it was confirmed that ROS‐sensitive hydrogel incorporating CGRP can be used to promote alveolar bone regeneration in diabetes‐associated periodontitis (**Figure** **9**).

At present, there is limited information regarding diabetic neuropathy in periodontium. The vast majority of studies on diabetic neuropathy focus on the skin, with only a few studies examining diabetic neuropathy in the femoral bone marrow.^[^ ^17^, ^26^ ^]^ In one of these studies, nerve fibers were observed 10 weeks after diabetes induction, and a negative correlation was found between the density of nerve fibers and bone loss.^[^ ^26^ ^]^ Therefore, in our study, db/db mice were euthanized at 12–14 weeks of age to identify potential neuropathy and to assess time‐dependent severity of the condition. This experimental design could be adapted to study diabetic neuropathy in various tissues and organs. Importantly, nerve endings have been demonstrated to play a role not just in skin wound healing and periodontitis, but also in pneumonia and inflammatory bowel diseases, among others.^[^ ^27^ ^]^ However, it remains unclear whether diabetic neuropathy exists in these organs and what the underlying impact of such neuropathy could be on these diseases.

NGF is an important neurotrophic factor in the processes of neurogenesis and neuron growth, and it can also promote the sprouting of peripheral CGRP^+^ nerve fibers.^[^ ^28^ ^]^ NGF expression has been shown to decrease in the serum and bone marrow of diabetic mice in some studies.^[^ ^17^, ^29^ ^]^ However, NGF expression was not altered in the diabetic periodontium based on our results. In addition to its influence on NGF, diabetes might affect CGRP^+^ sensory nerves by directly influencing sensory neurons in the trigeminal ganglia. According to a previous report, dorsal root ganglia, as sensory neurons, show no detectable alterations in short‐term experimental diabetes, while long‐term diabetic mice exhibit neuron perikaryal and distal axon atrophy with downregulation of CGRP transcription.^[^ ^30^ ^]^ However, obvious pathological changes and TUNEL labeling are not visible in the trigeminal ganglia in our study. Autophagy participates in maintaining the homeostasis of the intracellular environment, and severely impaired autophagy would lead to apoptosis. We detected autophagy flow within neurons in response to diabetic stress and found a significant decrease in autophagy in the diabetic trigeminal ganglion neurons. These results demonstrate that autophagy could be interfered at the early stage of diabetes, which might lead to apoptosis at a more advanced stage. The decreased content of CGRP in the trigeminal ganglia probably also resulted from the disrupted autophagy.

In this study, we mainly used the db/db mouse model to mimic the conditions of diabetes. This kind of type of mouse develops obesity and type 2 hyperinsulinemic diabetes.^[^ ^31^ ^]^ Notably, there are more functional abnormalities of sensory nerves in type 1 insulinopenic diabetes compared to type 2 diabetes.^[^ ^32^ ^]^ Specifically, the deficiency of insulin appears to be more important in nociceptive neuropathy than hyperglycemia. Therefore, it is highly possible that the current observations apply equally to type 1 diabetic mice.

The phenotypic similarities in the reduction of periodontal sensory nerve density and aggravated bone resorption observed in diabetes‐associated periodontitis and denervated periodontitis suggest a common pathophysiologic mechanism for defective bone tissue formation in states of peripheral neuropathy. It should be emphasized that diabetes is not the only cause of neuropathy. Chemotherapeutic agents such as paclitaxel and cisplatin can also lead to chemotherapy‐induced peripheral neuropathy, which affects up to 30 to 40% of patients undergoing treatment.^[^ ^33^ ^]^ Specifically, paclitaxel has been shown to disrupt microtubular stabilization of terminal dendrites to induce distal axonal degeneration,^[^ ^34^ ^]^ which leads to the dysfunction of sensory nerves. An in vivo study demonstrated that paclitaxel induces deficiencies in nerve regrowth and fracture healing. More importantly, cisplatin, as an antineoplastic agent, has been shown to exacerbate periodontitis.^[^ ^35^ ^]^ Thus, it is intriguing to speculate that chemotherapeutic agent‐induced peripheral neuropathy has a contributory role in periodontitis,^[^ ^36^ ^]^ but needs further validation.

Periodontium is richly innervated by sensory nerves, which can be labeled by SP and CGRP. However, the density of CGRP^+^ nerves was much higher than that of SP^+^ nerves in the present study. This finding is consistent with other studies’ findings.^[^ ^37^ ^]^ Additionally, the transcription level of CGRP is higher than that of SP in periodontium.^[^ ^38^ ^]^ In the trigeminal ganglia, CGRP^+^ neurons are also denser than SP^+^ neurons.^[^ ^39^ ^]^ In our study, the bone‐forming capacities in diabetic mice with periodontitis were significantly promoted by the local application of CGRP, without the involvement of other neurotrophins and neuropeptides. Hence, the influence of diabetes on periodontal sensory nerves is primarily reflected in the function of CGRP.

CGRP is a bridge between the musculoskeletal system and the nervous system. It promotes osteogenesis directly by affecting mesenchymal stem cells or indirectly by modulating blood vessels and immune reactions.^[^ ^40^ ^]^ We performed cell grouping by single‐cell analysis to identify CGRP‐target cells in the periodontium and found that endothelial cells are the major target cells. An ex vivo study demonstrated that the effect of CGRP on endothelial cell migration and tube formation is comparable to the critical angiogenesis regulator, vascular endothelial growth factor.^[^ ^41^ ^]^ In addition to the function of the vascular system in nutrient supply and waste removal, this system, especially type H vessels, can promote osteoblast differentiation by secreting high levels of Noggin and other molecules, which has been fully confirmed in the metaphysis region and sub‐periosteum of long bones.^[^ ^42^ ^]^ Specifically, Noggin, an important molecule in Notch signaling, can be secreted by endothelial cells to enhance the proliferation and differentiation of osteoprogenitors.^[^ ^43^ ^]^ Furthermore, exosomes derived from endothelial cells have been shown to promote the transformation of stem cells into the osteoprogenitor phenotype by increasing the expression of zinc finger and BTB domain‐containing 16.^[^ ^44^ ^]^ Hypoxia‐induced factor 1‐alpha (HIF‐1α) also plays a role in the paracrine signaling between endothelial cells and osteoprogenitors. This transcription factor is highly expressed in type H endothelial cells in young mice, but its expression gradually decreases with age, which is correlated with age‐related bone loss. The specific deletion of HIF‐1α in endothelial cells leads to a significant reduction in osteoprogenitors.^[^ ^45^ ^]^

Recently, a type H vessel was verified to be present in the periodontal ligament.^[^ ^23^ ^]^ Here, we observed more type H vessels in non‐diabetes‐associated periodontitis than in diabetes‐associated periodontitis. Several mechanisms may explain the reduction of type H vessels under diabetic conditions. High glucose levels directly impair endothelial cell proliferation, migration, and tube formation abilities by causing mitochondrial damage, showing a decreased number of mitochondrial autophagic vacuoles.^[^ ^46^ ^]^ In addition to high glucose, oxidative stress also contributes to vascular impairments, as diabetes leads to elevated levels of NADPH oxidase 1 and 2 (NOX 1 and 2) in endothelial cells. These impairments can be effectively alleviated by treatment with NOX2 inhibitors.^[^ ^47^ ^]^ Furthermore, our study demonstrated that CGRP can promote the formation of type H vessels and the subsequent aggregation of osteogenic precursor cells. The reduced density of CGRP^+^ nerves also accounts for the decreased density of type H vessels. It is important to emphasize that, unlike the changing pattern of CGRP^+^ nerves, the expression of VEGF in the periodontium is significantly higher in diabetes‐associated periodontitis patients compared to healthy individuals or patients with periodontitis alone.^[^ ^48^ ^]^ Therefore, the impaired formation of type H vessels in diabetes‐associated periodontitis is not due to a lack of VEGF but rather results from insufficient CGRP signaling.

Diabetes creates a severe microenvironment characterized by oxidative stress.^[^ ^49^ ^]^ Herein, an injectable ROS‐responsive hydrogel was prepared for CGRP delivery. CGRP is a sensory neuropeptide that consists of 37 amino acids without the quaternary structure of a protein. Therefore, CGRP would degrade rapidly if delivered directly as an exogenous substance.^[^ ^50^ ^]^ The use of hydrogel protects the loaded CGRP from proteolytic damage.^[^ ^51^ ^]^ Although CGRP^+^ nerves serve as nociceptive nerve terminals (also referred to as nociceptors) in the periodontium, and release of CGRP from sensory nerves can result in pain,^[^ ^52^ ^]^ the local application of CGRP@PVA/tsPBA hydrogels can promote bone regeneration without affecting pain levels in this article. This contradiction may be explained by the fact that the dose of CGRP required to modulate type H vessel formation is probably much lower than the concentrations that produce pain, and the hydrogels prevent an immediate burst of CGRP release after delivery. It is important to note that although the free‐radical‐scavenging ability of the PVA/tsPBA hydrogels was observed in vitro, the in vivo study showed no significant difference between the NC group and the PVA/tsPBA group. This outcome may be due to the consistently high concentration and sustained production of ROS in diabetes‐associated periodontitis,^[^ ^53^ ^]^ which likely exceeds the hydrogel's capacity to reduce ROS to levels that slow bone resorption and promote bone formation. Similar findings have been reported in multiple studies on PVA/tsPBA hydrogels, where no notable differences between control and blank hydrogel groups were observed in animal studies.^[^ ^51^, ^54^ ^]^ In line with these studies, our PVA/tsPBA hydrogels were primarily utilized for dynamic drug delivery and are therefore referred to as ROS‐responsive rather than ROS‐scavenging hydrogels.

## 4 Conclusion

The present study reveals the importance of the sensory nervous system for alveolar bone regeneration in diabetes‐associated periodontitis, using the db/db and periodontal denervated mouse models. Notably, CGRP promotes bone healing via the activation of type H vessel formation. Subsequently, we have developed the injectable ROS‐responsive PVA/tsPBA hydrogels to encapsulate CGRP for in situ delivery. Indeed, this delivery system could favourably modulate the neural microenvironment in periodontium under diabetic conditions in response to ROS stimulation. Collectively, the activation of type H vessel formation by CGRP may be an innovative therapeutic strategy to enhance alveolar bone healing in patients with diabetes‐associated periodontitis.

## 5 Experimental Section

### Experimental Animals

All the animal surgical procedures were carried out strictly according to the protocol approved by the HKU Ethics Committee, Committee on the Use of Live Animals in Teaching and Research (CULATR) (CULATR 23–552). Specific pathogen‐free (SPF) eight‐week‐old male db/+ and db/db mice (B6.BKS‐Leprdb) were acquired from the Wuhan Boster Biological Technology, Ltd (Wuhan, China). The mice were kept in an SPF animal facility and provided with a regular lab diet and water. A One‐Touch glucometer was used to measure blood glucose levels through the tail vein. The weights of mice were recorded weekly from 9 to 14 weeks of age. In addition, eight‐week‐old male C57BL/6J mice were also used under SPF conditions for unilateral sensory denervation surgery.

### Construction of a Mouse Model of Experimental Periodontitis

Ketamine and xylazine, two commonly used agents in animal anesthesia, produce acute hyperglycemia. In contrast, pentobarbital does not affect blood glucose levels.^[^ ^55^ ^]^ Therefore, in this study, mice were deeply anesthetized with 0.3% pentobarbital at a dose of 20 mL kg^−1^ intraperitoneally. A 5‐0 silk suture was tied around the left upper second molars of 14‐week‐old male db/+ and db/db mice, and the right side was used as a control counterpart to get the baseline of the alveolar crest. To compare the bone formation capacities of CGRP‐loaded hydrogels and blank hydrogels, both sides of the maxillary second molar were ligatured (Figure S9, Supporting Information). A 5‐0 silk ligature was also tied around the bilateral mandibular first molar of C57BL/6J mice on the 5th day after unilateral IAN axotomy (Figure S10, Supporting Information). The ligatures were retained for 7 days to induce periodontitis.

### IAN Axotomy

Under deep anesthesia, an extraoral horizontal cut was made on the skin to expose the masseter muscle, which was then precisely incised to reach the mandibular ramus. A quarter‐round carbide bur was employed to remove the cortical bone of the mandible, exposing the main trunk of IAN. ≈1 mm of the nerve was excised near the mandibular foramen, avoiding injury to inferior alveolar vessels. In the end, muscle and skin were closed and stitched with absorbable and non‐absorbable 6‐0 silk sutures, respectively (Figure 4A). All surgeries for sensory denervation were carried out on the left side, while the sham operation took place on the right side. Except for the nerve resection, the sham operation was conducted in the same way as the IAN axotomy. To verify the success of the operation, mechanical touch threshold measurements on the lower lips were conducted based on a previous study,^[^ ^56^ ^]^ and the CGRP‐positive nerve density in periodontal tissues was detected using immunofluorescence staining before the start of the experiments.

### Tissue Collection and Micro‐CT Analysis

Following transcardial perfusion using 0.9% saline solution and 4% paraformaldehyde, the maxillary samples and mandibular samples, as well as the trigeminal ganglia, were collected for further analysis. Micro‐CT (µCT 80, Scanco Medical, AG, Switzerland) was used to examine the morphological changes in the alveolar bone. The scan conditions were as follows: isotropic voxel size of 10.4 µm, X‐ray voltage of 55 kV, current of 145 µA, and integration time of 230 ms. 3D image generation and measurements were performed using Mimics 14.11 software (Materialize Corp., Leuven, Belgium), CTAn, and DataViewer (SkyScan, Kontich, Belgium) by a blind observer. Briefly, the measurements of bone volume/total volume (BV/TV) and bone density in the furcation area were performed after selecting a 3D region of interest (ROI) from the sagittal dataset. The ROI was extended from the mesial root to the distal root of the ligated molar. The distance between the cementoenamel junction (CEJ) and the alveolar bone crest (ABC) was assessed using 3D models, and the bone loss of the molar was measured at six points, including buccal‐mesial (B‐M), buccal‐middle (B‐MD), buccal‐distal (B‐D), palatal or lingual‐mesial (P/L‐M), palatal or lingual‐middle (P/L‐MD), and palatal or lingual‐distal (P/L‐D). Changes in BV/TV, bone density, and bone height were calculated by subtracting the value at the corresponding contralateral site from the measured value.

### Histological and Immunofluorescence (IF) Staining

Following micro‐CT scanning, the bone tissues were fully decalcified over a period of 4 weeks using 0.5 mol L^−1^ EDTA at 4 °C. For sectioning at 4 µm thickness, the ganglia and decalcified tissues were embedded in paraffin. Histological morphology was assessed using haematoxylin and eosin (H&E) staining, along with Masson's trichrome staining. The TRAP kit (387A, Sigma‐Aldrich) was used to stain osteoclasts. The TUNEL assay was used to examine apoptosis (C1090, Beyotime). The stained slides were observed and captured using a Nikon Eclipse LV100 POL microscope. Quantification of the osteoclasts was performed using ImageJ. Additionally, the sections underwent antigen retrieval treatment with Tris‐EDTA buffer. Following a block with 10% goat serum, the sections were exposed to primary antibodies overnight at 4 °C. Antibody information is listed in Table S1 (Supporting Information). Following this, the sections were treated with the appropriate secondary antibodies for 60 min. Positive controls are shown in Figure S11 (Supporting Information). For negative controls, only secondary antibodies were applied, and nuclei were counterstained using a mounting medium that included DAPI (ab104139, Abcam).

All the quantifications were conducted blindly. For quantifying osteoclasts and Osterix^+^ cells, four visual fields were randomly selected. Then, the cell count was directly performed by the Cell Counter plugin in ImageJ, and the average number of cells across these fields was taken. The quantification of type H vessels was also conducted using ImageJ in four randomly chosen visual fields, wherein the critical step involved manually adjusting the threshold to separate the yellow‐stained type H vessels from the background. After defining criteria for minimum size and maximum pixel area to exclude non‐target objects from the analysis, the particle analysis was automated using plugins. Nerve fibers were quantified with the help of an ImageJ plug‐in called VesselJ, applied to four randomly selected visual fields.^[^ ^56^ ^]^ The intensity of immunofluorescence was directly measured using the ZEN 3.4 software (Zeiss).

To determine whether a single section can accurately represent the density of peripheral nerves, IF staining was performed for β 3 Tubulin on three serial sections at 10‐slide intervals. The nerve density values from three sampled sections of each animal were averaged, and this mean was considered the final value of that animal. It is found that the observed trends were consistent throughout the periodontal tissues (Figure S12, Supporting Information).

To further strengthen the completeness of the results, additional images of the alveolar bone crest obtained by H&E staining, Masson's trichrome staining, TRAP staining, and IF staining are presented in Figures S13–S15 (Supporting Information).

### Single‐Cell RNA‐Sequencing Data Re‐Analysis

The single‐cell RNA‐sequencing (scRNA‐seq) datasets GSE164241 and GSE188217 were downloaded from the GEO database. The former dataset contained 13 normal gingival mucosa samples and 8 gingival mucosa samples from periodontitis individuals. The latter dataset involved gingival tissues from db/db mice and db/+ mice. The “Seurat” package (ver. 5.1.0) was used to preprocess and transform the data. Genes with over 5000 features per cell, cells with less than 200 unique features, and cells with a mito percent exceeding 15% were removed from the human data. In the mouse data, genes expressed in less than three cells, cells with fewer than 300 gene expressions, and cells with a mito percent of more than 25% were filtered out. Then, the DoubletFinder package was used to identify and remove doublets. Finally, the scRNA‐seq data of healthy human gingival tissues and inflammatory human gingival tissues included 49802 cells and 27314 cells, respectively, while the single‐cell transcriptomes of 6469 cells and 10869 cells were obtained from db/+ mice and db/db mice. The batch effect was removed using the R package Harmony. The variable genes were selected by the FindVariableGenes function in the Seurat package. Based on PCA outcomes, UMAP was applied for dimension reduction and visualization. Human cell types were predicted through manual annotation using ACKR1, PECAM1, RAMP2, SELE, and VWF to identify endothelial cells; COL1A1, COL3A1, DCN, LUM, and CFD to identify fibroblasts; CD52, CD69, CXCR4, HCST, and PTPRC to identify immune cells; and FABP5, KRT5, KRT14, SPRR1B, and DSP to identify epithelial cells. Mouse cell type annotation was also performed manually using Ackr1, Pecam1, Ramp2, Sele, and Vwf to identify endothelial cells; Col1al, Col3a1, Dcn, and Lum to identify fibroblast; Cd52, Cd69, Cxcr4, Hcst, and Ptprc to identify immune cells; and Fabp5, Krt5, Krt14, Lgals7, and Dsp to identify epithelial cells. The expression of marker genes in each cell type was represented as Dotplots (Figure S16, Supporting Information). Levels of CGRP receptor‐related gene expression were visualized by VlinPlot using the R package MySeuratWrappers.

### Synthesis of tsPBA Linker and Construction of PVA/tsPBA Hydrogels

In order to synthesize the tsPBA linker, 2 g (9.2 mmol) of 4‐(bromomethyl)phenylboronic acid (BMPBA) was first dissolved in 40 mL of dimethylformamide, and subsequently, 0.4 g (3.0 mmol) of N,N,N′,N′‐tetramethyl‐1,3‐propanediamine (TMPA, 0.4 g, 3.0 mmol) was introduced. After stirring at 60 °C for 24 h, the supernatant was filtered, followed by adding into 100 mL of tetrahydrofuran (THF) to obtain a white solid precipitate. Next, at room temperature, centrifugation of the suspension was performed at 1500 g for 5 min, and the precipitation was collected and washed with THF (20 mL) for 3 times. After drying under a vacuum overnight, pure tsPBA was obtained (yield ≈ 70%). The structure of tsPBA was identified by dissolving the obtained powder in deuterated DMSO and examining it by proton nuclear magnetic resonance (1H NMR) spectroscopy on a 400 MHz device.

PVA (106 kDa; 98–99% hyfrolyzed) was dissolved in deionized water at 90 °C to obtain a PVA solution with a concentration of 3 wt.%. PVA/tsPBA hydrogels were instantly formed by mixing equal volumes of tsPBA solution (3 wt.%) and PVA solution (3 wt.%) through a dual syringe. To load cargo into the hydrogel, CGRP (HY‐P0203A, MCE) was resuspended in the tsPBA solution to achieve a CGRP concentration of 10 µM, followed by the addition of PVA to form the hydrogel. The selection of CGRP concentration was based on previous literature.^[^ ^12^ ^]^

### Scanning Electron Microscopy (SEM) Observation

The internal structures of PVA/tsPBA hydrogels and CGRP@PVA/tsPBA hydrogels were observed by SEM (Hitachi S‐4800) with a voltage of 10 kV. Specifically, the hydrogels were frozen and fractured at −80 °C, followed by freeze‐drying to remove the water. Then, the hydrogels were coated with gold to improve their conductivity.

### Rheological Experiment, Swelling Rate, and Adhesion Test of Hydrogels

The rheological properties of the hydrogels were assessed using a HAAKE MARS rheometer (Thermo Scientific) equipped with 20 mm parallel plates. Experiments were conducted at 25 °C, frequency 0.1 to 10 rad s^−1^, strain 1%, and the energy storage modulus (G′) and loss modulus (G″) were recorded.

Swelling experiments were conducted in PBS at 37 °C. The starting weights of the freeze‐dried hydrogels (*n* = 3) were recorded as W_d_. At different time points, swollen hydrogels were obtained. After blotting off the surface water with filter paper, the hydrogels' weights were recorded as W_t_. The swelling ratio was determined by the formula: swelling ratio = (W_t_ − W_d_)/W_d_.

In addition, the adhesion properties of the PVA/tsPBA hydrogel were evaluated using a lap shear test. A solution of 0.1 mL of PVA and tsPBA was injected simultaneously to directly form the hydrogel on a glass plate, which was then covered with another glass plate. The bond strength was measured using a texture analyzer (Rapid TA+) at a tensile rate of 1.0 mm s^−1^.

### Biocompatibility Tests of Hydrogels

Human umbilical vein endothelial cells (HUVECs) were grown at a density of 5000 cells per well in 96‐well plates overnight to enable adhesion. To assess the biocompatibility of the hydrogels, suitable amounts of PVA/tsPBA hydrogels were weighed and placed in pure complete DMEM medium, with the hydrogel concentration reaching 100 mg mL^−1^. The medium with PVA/tsPBA was then incubated at 37 °C for 48 h to obtain a hydrogel extract medium. This extract medium was added to HUVECs in different concentrations for 1 day. Finally, cell viability assessment of the HUVECs was performed using a CCK8 assay kit.

### Degradation of Hydrogels and Drug Release Analysis

Hydrogels with a diameter of 8 mm and a length of 3 mm were used. Samples were immersed in PBS (3 mL) or PBS with H_2_O_2_ (1 mM) at room temperature.^[^ ^51^ ^]^ At 0, 5, 10, and 15 days, images were taken of the remaining hydrogels. To examine the effect of H_2_O_2_ on the hydrogel structure, SEM images were taken of the hydrogel incubated with 1 mM H_2_O_2_ at 0, 5, and 10 days.

The ROS‐scavenging capability of the PVA/tsPBA hydrogel was determined using a hydrogen peroxide concentration kit (S0038, Beyotime). Briefly, the hydrogels were weighted and immersed in a freshly prepared 1 mM H_2_O_2_ solution at a 10 mg mL^−1^ ratio. At each time point, 200 µL of the solution was obtained. Then, the same volume of fresh solution was replaced in the sample. According to the manufacturer's protocol, the hydrogen peroxide concentration was measured to evaluate the scavenging ability of PVA/tsPBA hydrogel.

To determine the drug release profile, 200 µL CGRP@PVA/tsPBA hydrogels were immersed in a H_2_O_2_ solution (1 mM, 200 µL) at 37 °C. At the designated time point, all of the supernatant was obtained, and the same volume of fresh solution was replaced in the sample. Then, High‐Performance Liquid Chromatography (HPLC) was used to directly evaluate the release profile of the CGRP peptide. Column information: Agilent InfinityLab Poroshell 120 EC‐C18 column (100 mm × 4.6 mm, 2.7 µm particle diameter), with Poroshell 120 UHPLC Guard column; Detection wavelength: 220 nm.

### Evaluation of Loaded CGRP Structural Stability

To determine whether the PVA/tsPBA hydrogels can protect the loaded biomolecule from proteolytic degradation, the hydrogel‐loaded CGRP and unencapsulated CGRP were detected by Western Blot after enzymatic digestion. Briefly, CGRP@PVA/tsPBA hydrogels (3 mm in length and 8 mm in diameter) were prepared and added into trypsin or proteinase K protease solutions at various concentrations. As a control, the same amount of CGRP was directly exposed to the proteinase. After 20 min of proteolytic degradation, a HALTTM protease inhibitor cocktail and phosphatase inhibitor cocktail (1 861 281, Thermo Scientific) were added. In addition, the hydrogels were fully minced by scissors and treated with an H_2_O_2_ solution to release loaded CGRP. Finally, the western blot analysis was performed routinely with CGRP antibody (ab283568, Abcam) and polyvinylidene difluoride membrane (Immobilon PVDF, 0.2 µm, Millipore).

### In Vivo Treatments of CGRP@PVA/tsPBA Hydrogels

The bilateral periodontitis model was first established in db/db mice, and the ligatures were removed after 7 days. Hydrogels were directly injected into periodontal pockets using dual syringes filled with 10 µL of PVA and 10 µL of CGRP‐loaded tsPBA solutions every 7 days (Figure S17, Supporting Information). In contrast, the PVA/tsPBA group was established by injecting 10 µL of PVA and 10 µL of tsPBA solutions into the periodontal pockets without the addition of CGRP. Additionally, to assess the effect of unloaded hydrogels on tissue regeneration, a negative control (NC) group was set up. At 28 days after the injection of the hydrogel, all mice were euthanized to harvest their maxillae for the follow‐up experiments.

### Spontaneous Pain‐Like Behavior Testing

Three groups of mice were tested one day before and one day after the removal of the ligature and the injection of hydrogels. Spontaneous pain‐like behavior was recorded for 30 min, beginning 15 min after the animal subject was placed in a transparent plastic cubic cage (15 × 15 × 15 cm), using the mouse grimace scale (MGS) and face grooming as measures. To obtain mean MGS scores, one clear facial image per mouse was taken every three minutes of video. The scores for the five facial action units, including cheek bulge, nose bulge, whisker change, ear position, and orbital tightening, were summed and averaged to calculate the mean MGS score.^[^ ^57^ ^]^ A stopwatch was used to measure the total time spent by the mice on face grooming episodes, as previously described.^[^ ^58^ ^]^ To calculate the difference of score or time, baseline scores and times obtained one before the injection of hydrogels were subtracted from the scores and times obtained one day after the operation. All analyses were performed by an experimenter who did not know the experimental protocols.

### Statistical Analysis

The data were presented as means ± standard deviation (SD). SPSS 25.0 software was used to conduct the analysis. Differences between the db/+ and the db/db groups were tested using a two‐tailed unpaired *t*‐test or nonparametric *t*‐test, as appropriate. To assess the differences between the operated and sham‐operated sides, either a two‐tailed paired *t*‐test or a paired nonparametric *t*‐test was employed, depending on the situation. To determine the differences among the control group, PVA/tsPBA group, and CGRP@PVA/tsPBA group, one‐factor analysis of variance (ANOVA) was performed, followed by the Least Significant Difference (LSD) test or Kruskal‐Wallis H test with Dunn‐Bonferroni post hoc test, as appropriate. ANOVA with LSD test was also utilized to measure differences for more than two groups in characterizing the properties of hydrogels. One‐way repeated measures ANOVA was used for drug release profile analysis. Statistical significance was set at *p* < 0.05, and *n* represents the number of animals.

## Conflict of Interest

The authors declare no conflict of interest.

## Supporting information



Supporting Information

## Data Availability

The data that support the findings of this study are available from the corresponding author upon reasonable request.
