# Supplementary material for: CGRP‐Loaded ROS‐Responsive Hydrogel Restores Neuro‐Angiogenic Signaling to Promote Bone Regeneration in Diabetes‐Associated Periodontitis
Source: Adv Sci (Weinh). 2025 Aug 4;12(40):e06438. doi: 10.1002/advs.202506438 (PMC12561402; doi:10.1002/advs.202506438)
Supplement: Supplementary file 1 — Supporting Information [file ADVS-12-e06438-s001.docx]

Supporting Information

Title

CGRP-Loaded ROS-Responsive Hydrogel Restores Neuro-Angiogenic Signaling to Promote Bone Regeneration in Diabetes-Associated Periodontitis

Chaoning Zhan, Qingyi Dai, Jianhan Ren, Lijian Jin, Weiping Wang, Zhou Ye, James Kit Hon Tsoi, Yifan Lin*

**Table S1.** Antibodies used in this study

| Antibodies | Source | Identifier |
| --- | --- | --- |
| Anti-CGRP | Abcam | Cat#ab283568 |
| Anti-β 3 Tubulin | Abcam | Cat# ab78078 |
| Anti-SP | Abcam | Cat# ab14184 |
| Anti-CD31 | Abcam | Cat# ab281583 |
| Anti-EMCN | Abcam | Cat# ab106100 |
| Anti-OPG | Abcam | Cat# ab183910 |
| Anti-RANKL | Abcam | Cat# ab216484 |
| Anti-OSX | Abcam | Cat# ab209484 |
| Anti-CRCP | Abcam | Cat# ab139264 |
| Anti-Rabbit IgG H&L (Alexa Fluor^®^ 488) preadsorbed | Abcam | Cat#ab150081 |
| Anti-Rat IgG H&L (Alexa Fluor^®^ 568) preadsorbed | Abcam | Cat# ab175710 |
| Anti-mouse IgG H&L (Alexa Fluor^®^ 647) preadsorbed | Abcam | Cat# ab150119 |
| Anti-NGF | Bioss | Cat# bs-0067R |


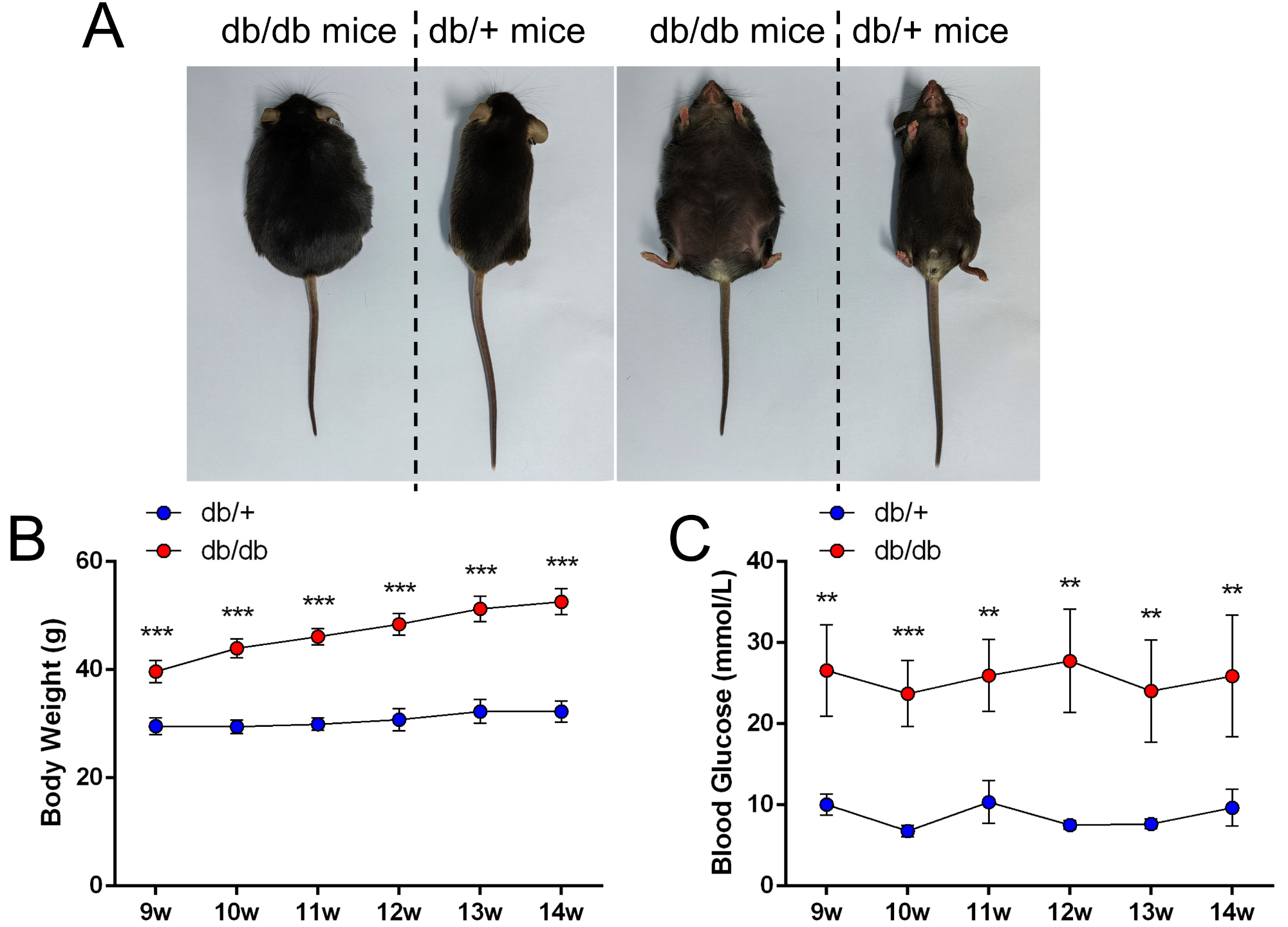


**Figure S1.** Photograph (A), body weight (B), and blood glucose levels (C) in db/+ and db/db mice (n = 4). ^**^ *P* < 0.01, ^***^ *P* < 0.001.


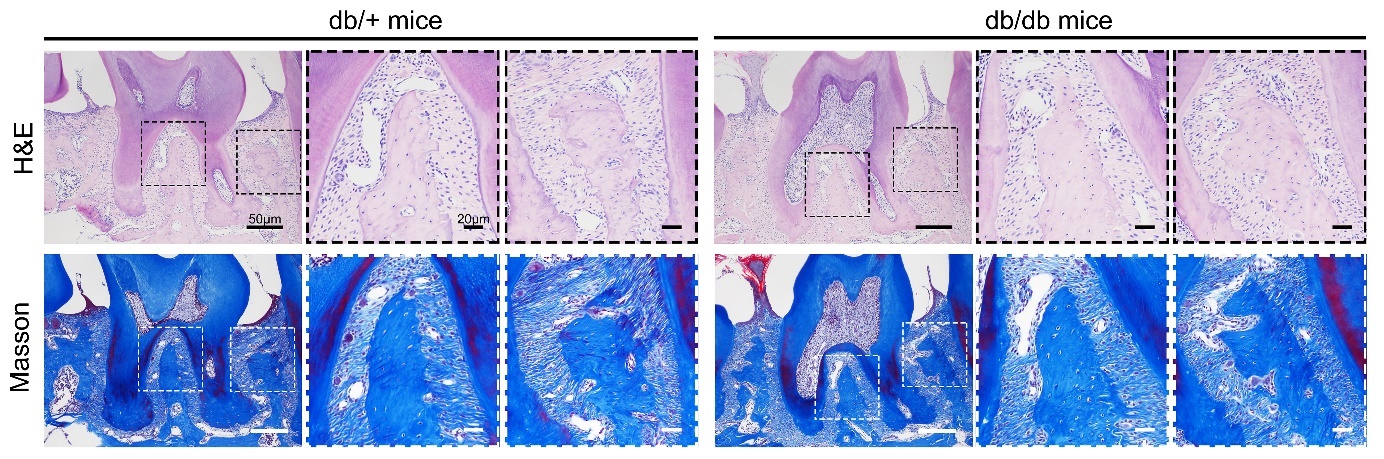


**Figure S2.** H&E staining and Masson’s trichrome staining of periodontal tissues in 14-week db/+ mice and db/db mice. Left boxes: furcation area; Right boxes: alveolar bone crest.


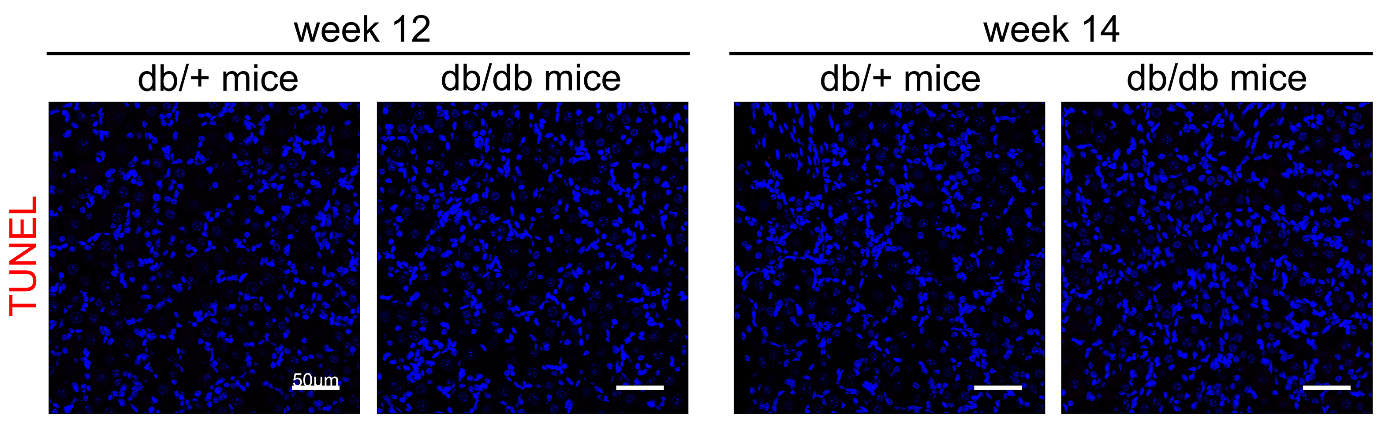


**Figure S3.** Representative images of terminal deoxynucleotidyl-transferase-mediated dUTP nick end labeling (TUNEL) staining (red) of the trigeminal ganglia.


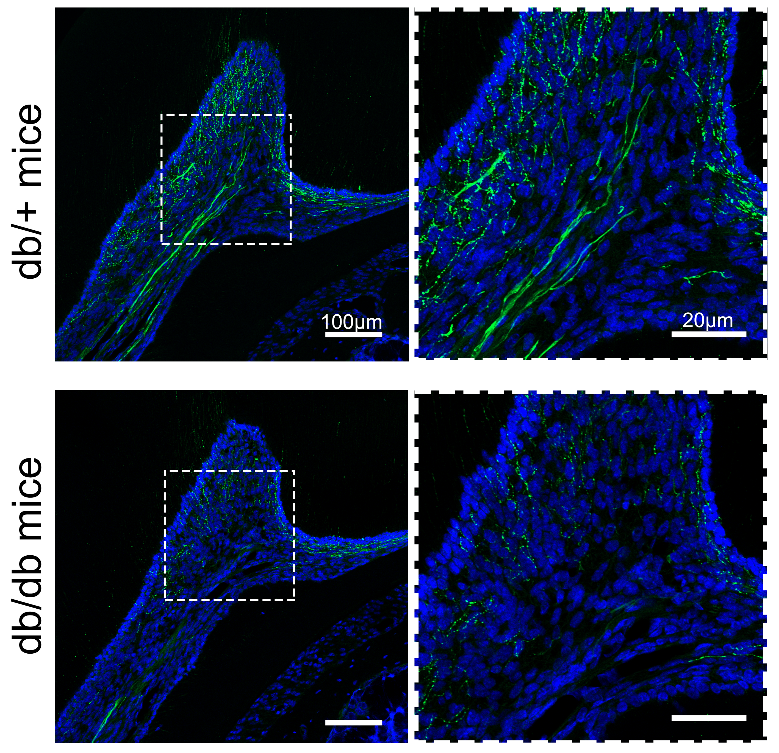


**Figure S4.** Calcitonin gene-related peptide-positive nerves (green) in the dental pulp of the first molars in db/+ mice and db/db mice.


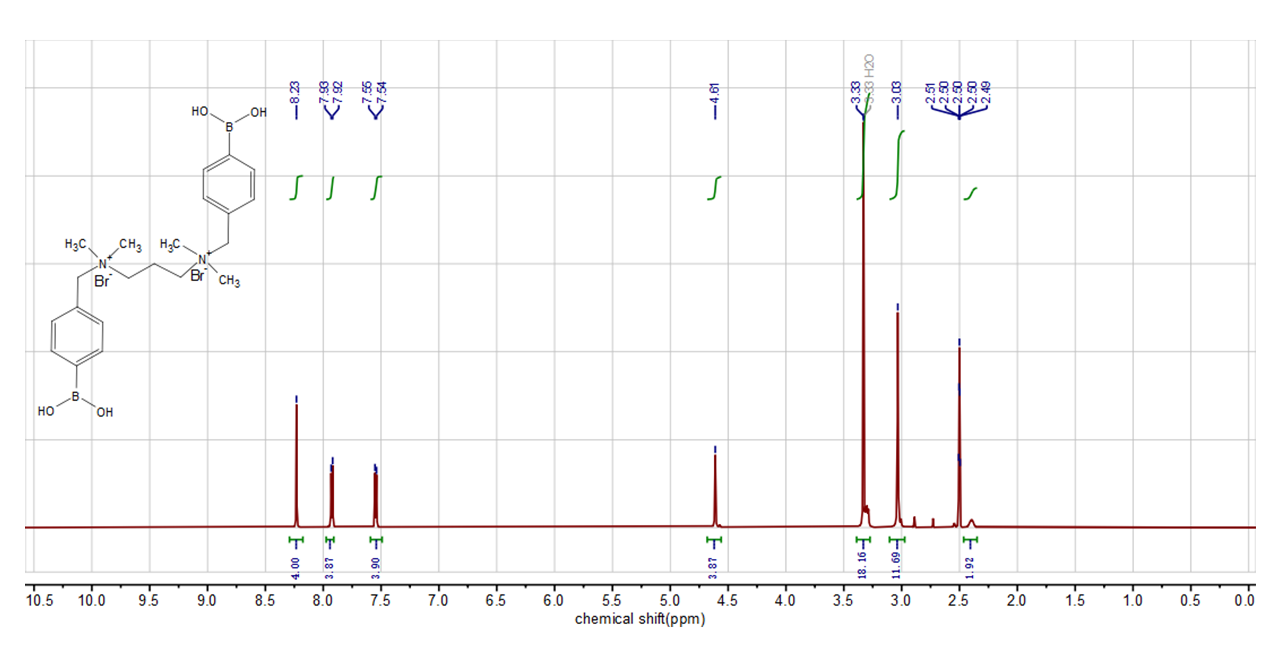


**Figure S5.** ^1^H NMR spectrum of tsPBA.


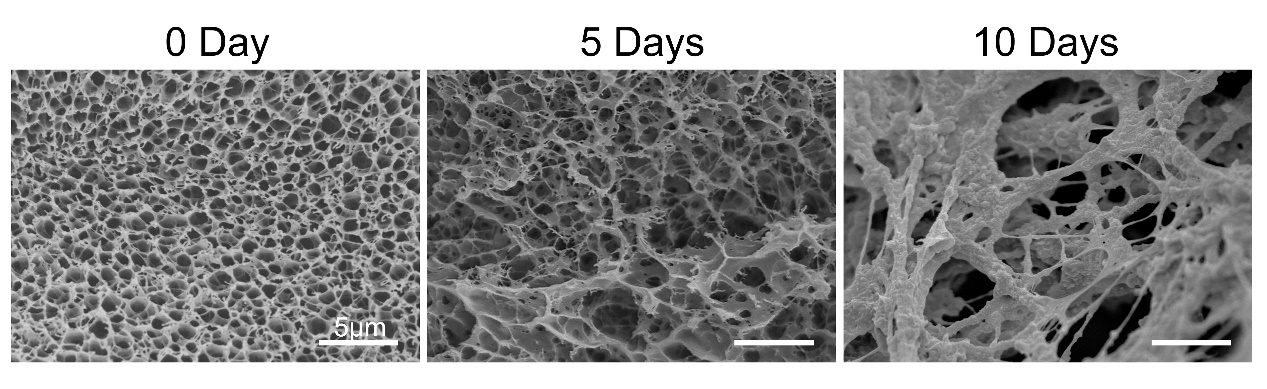


**Figure S6.** Representative scanning electron microscopy images of the PVA/tsPBA hydrogels incubated in an H_2_O_2_ solution at various time points (all images at same magnification).


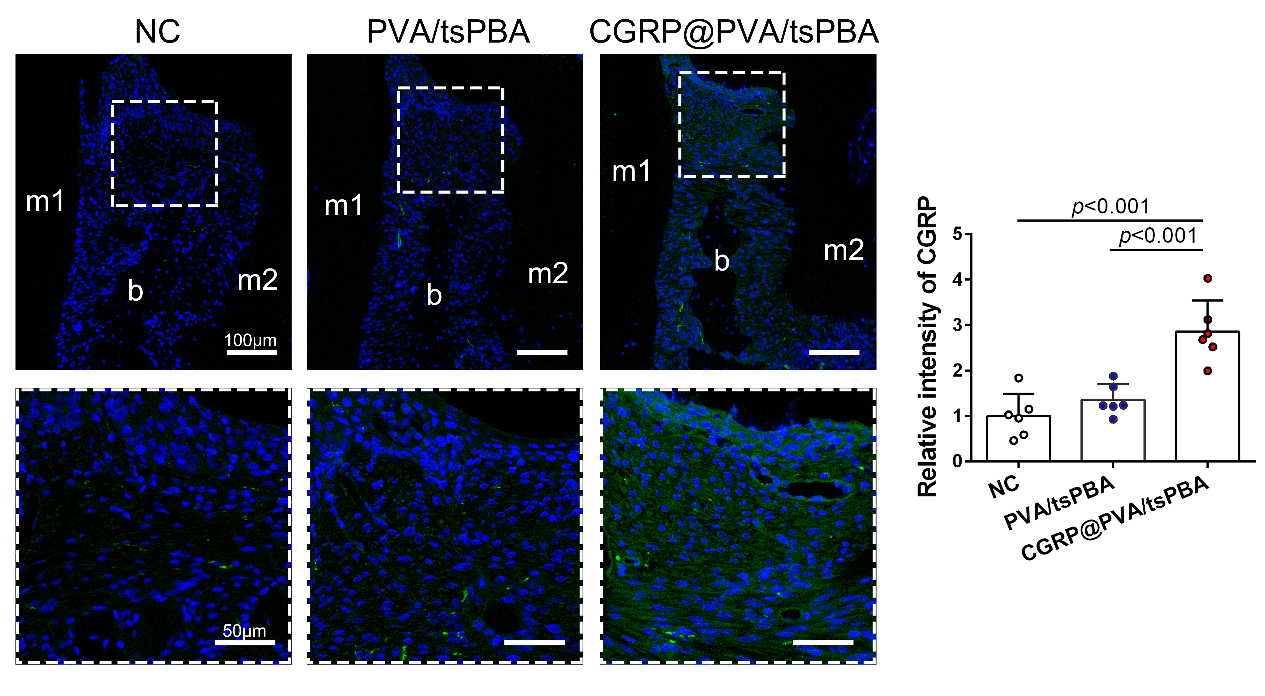


**Figure S7.** Immunofluorescence staining and quantification of CGRP levels in periodontium following hydrogel injection (green). b, bone; m1, the first molars; m2, the second molars.


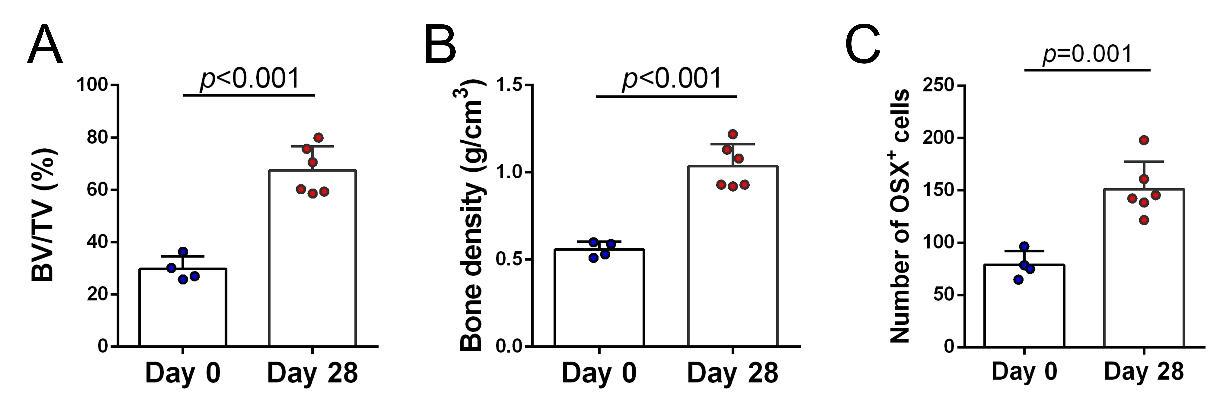


**Figure S8.** The application of CGRP@PVA/tsPBA hydrogels promoted the aggregation of osteoprogenitors and exerted regenerative effects on alveolar bone tissues. A and B) The hydrogels increased the bone volume/tissue volume ratio and bone density in the root furcation area (n = 4 or 6). C) The hydrogels accelerated the generation of new OSX^+^ cells in the periodontium. OSX, Osterix.


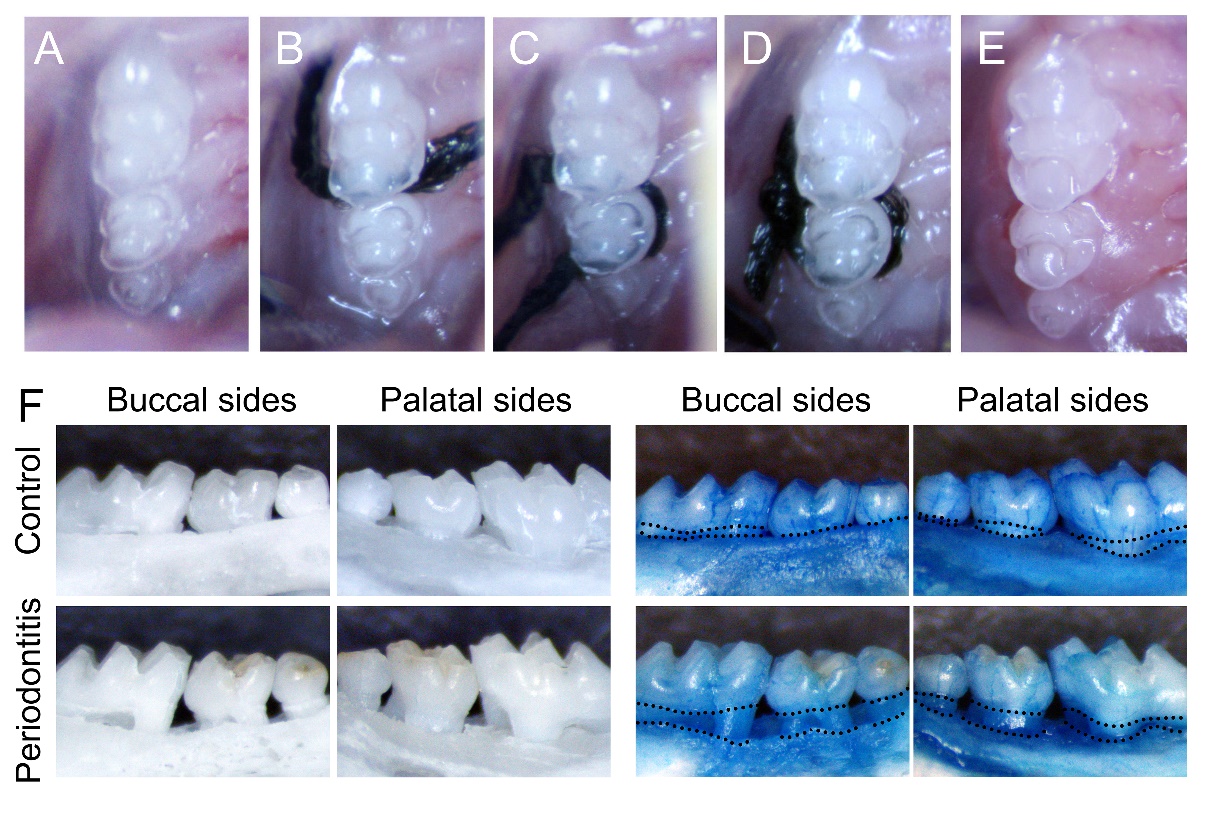


**Figure S9.** Procedures for the construction of a maxillary ligature-induced periodontitis mouse model and the representative images of the bone loss in the control and periodontitis groups. A-D) The procedures for ligation placement. E) A photograph taken 7 days after ligation placement. F) The buccal and palatal sides in control and periodontitis groups, with or without methylene blue staining.


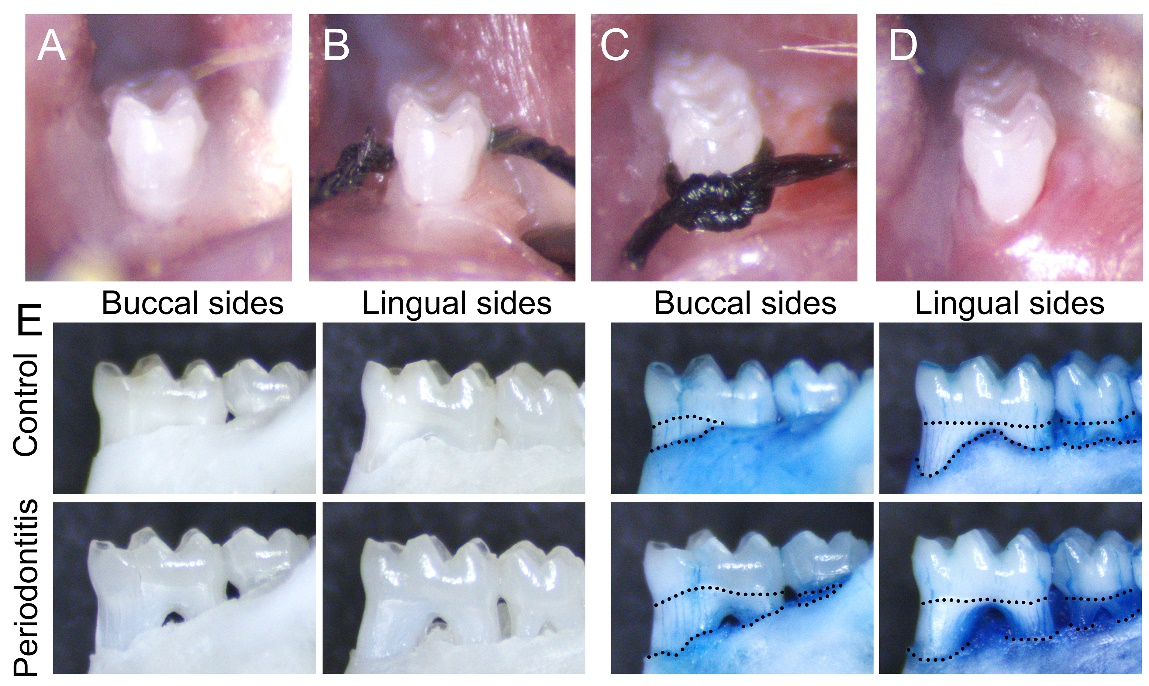


**Figure S10.** Procedures for the construction of a mandibular ligature-induced periodontitis mouse model and representative images of the bone loss in control and periodontitis groups. A-C) The procedures for ligation placement. D) A photograph taken 7 days after ligation placement. E) The buccal and lingual sides in control and periodontitis groups, with or without methylene blue staining.


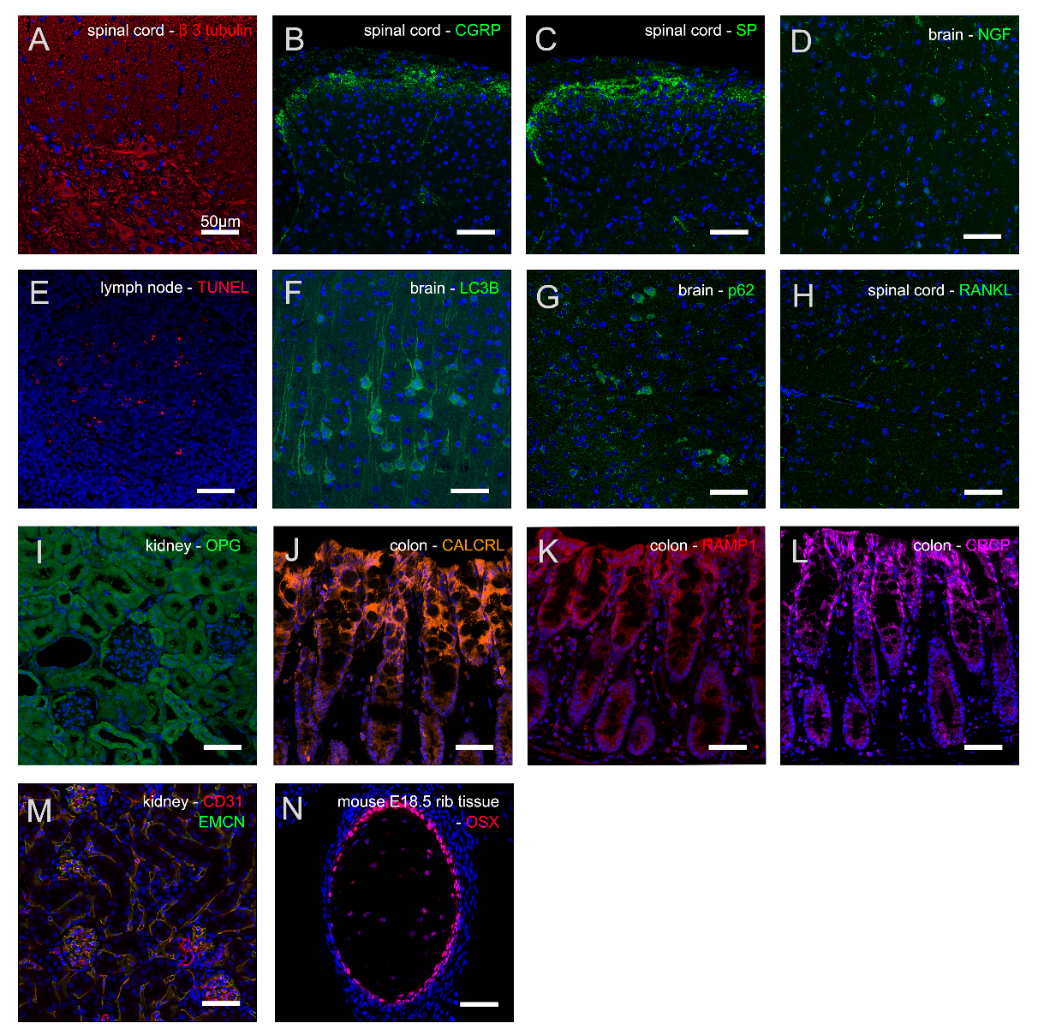


**Figure S11.** Results obtained from positive samples. IF analysis of paraffin-embedded mouse: A) spinal cord tissue labeling β 3 tubulin primary antibody, B) spinal cord tissue labeling CGRP primary antibody, C) spinal cord tissue labeling SP primary antibody, D) brain tissue labeling NGF primary antibody, E) TUNEL apoptotic cell detection in lymph node tissue section, F) brain tissue labeling LC3B, G) brain tissue labeling p62, H) spinal cord tissue labeling RANKL primary antibody, I) kidney tissue labeling OPG primary antibody, J) colon tissue labeling CALCRL, K) colon tissue labeling RAMP1, L) colon tissue labeling CRCP primary antibody, M) kidney tissue labeling CD31 and EMCN primary antibody, and N) rib tissue of mouse E18.5 labeling OSX primary antibody. CALCRL, calcitonin receptor-like receptor; CGRP, calcitonin gene-related peptide; CRCP, CGRP receptor component protein; EMCN, endomucin; IF, immunofluorescence; LC3B, microtubule-associated protein 1 light chain 3 beta; NGF, nerve growth factor; OPG, osteoprotegerin; OSX, Osterix; RAMP1, receptor activity-modifying protein 1; RANKL, receptor activator of NF-κB ligand; SP, substance P.


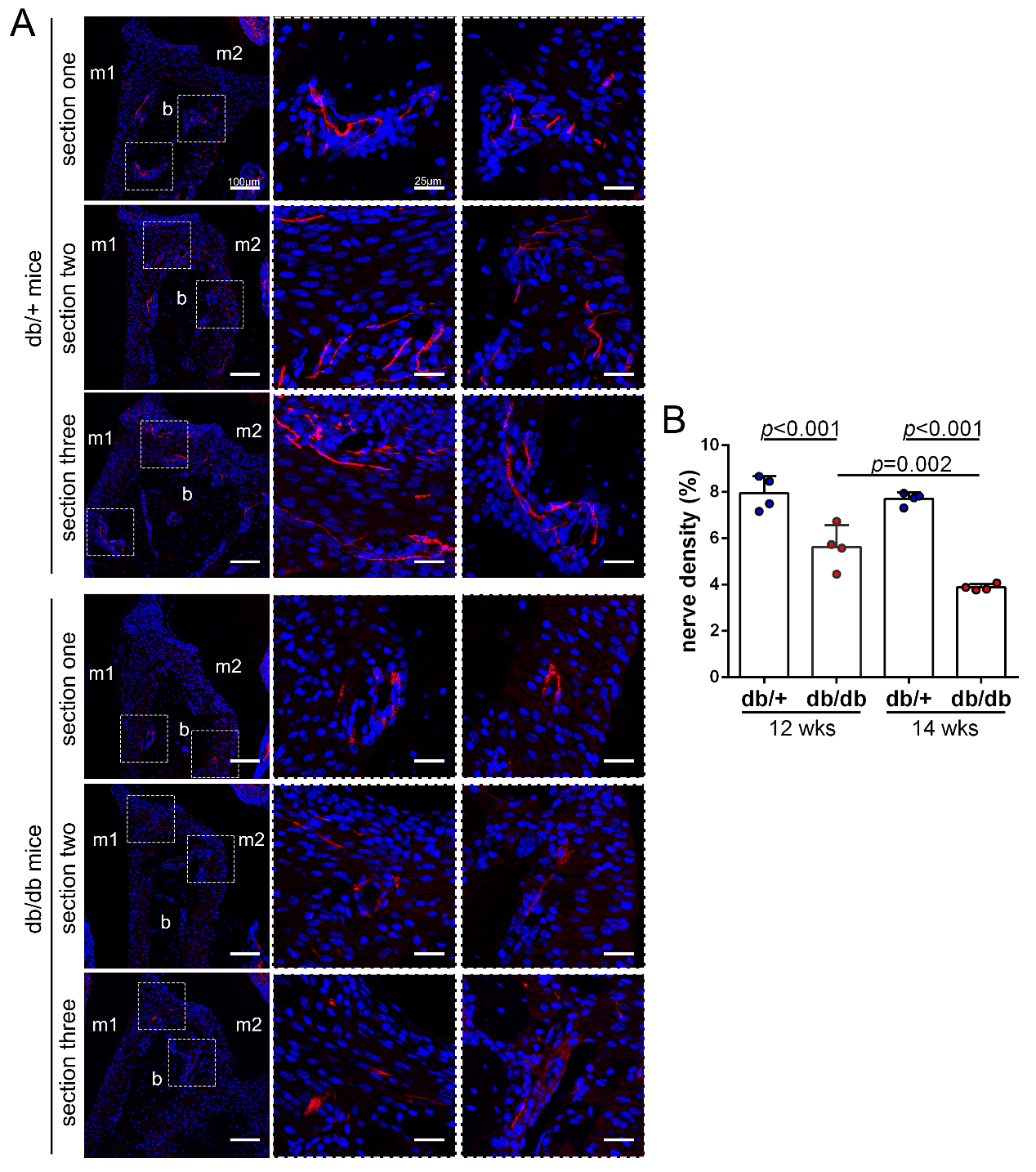


**Figure S12.** Serial sections illustrating the loss of periodontal nerves in db/db mice. A) Immunofluorescence staining revealing changes in the density of β 3 Tubulin^+^ nerves (red). B) Quantitative analysis of β 3 Tubulin^+^ nerve density confirming a lower density in the periodontal tissues of db/db mice compared to db/+ mice (n = 4). b, bone; m1, the first molars; m2, the second molars.


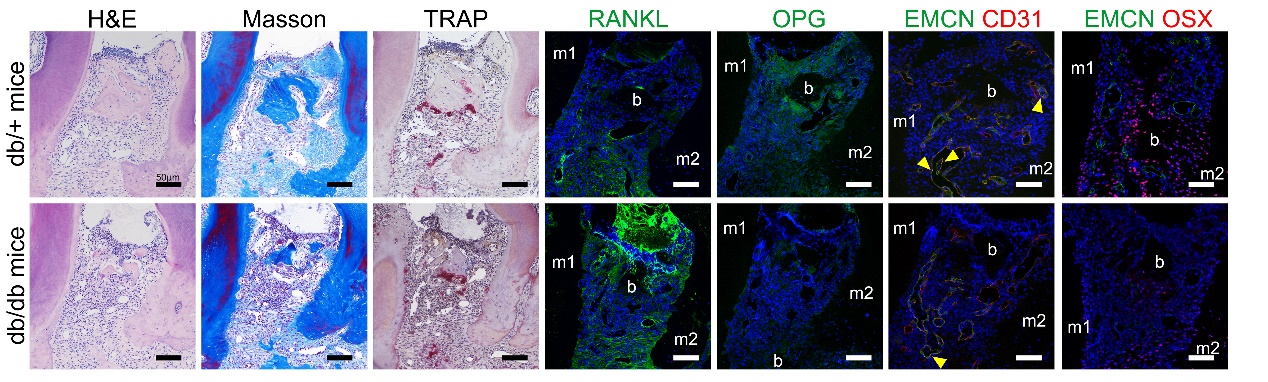


**Figure S13.** H&E staining, Masson's trichrome staining, TRAP staining, and immunofluorescence staining demonstrated aggravated bone loss, increased numbers of osteoclasts, higher expression of RANKL, lower expression of OPG, reduced density of type H vessels, and fewer OSX-positive osteoprogenitors in the alveolar bone crest of db/db mice. OPG, osteoprotegerin; RANKL, receptor activators of nuclear factor kappa-B ligand; EMCN, endomucin; OSX, Osterix. b, bone; m1, the first molars; m2, the second molars.


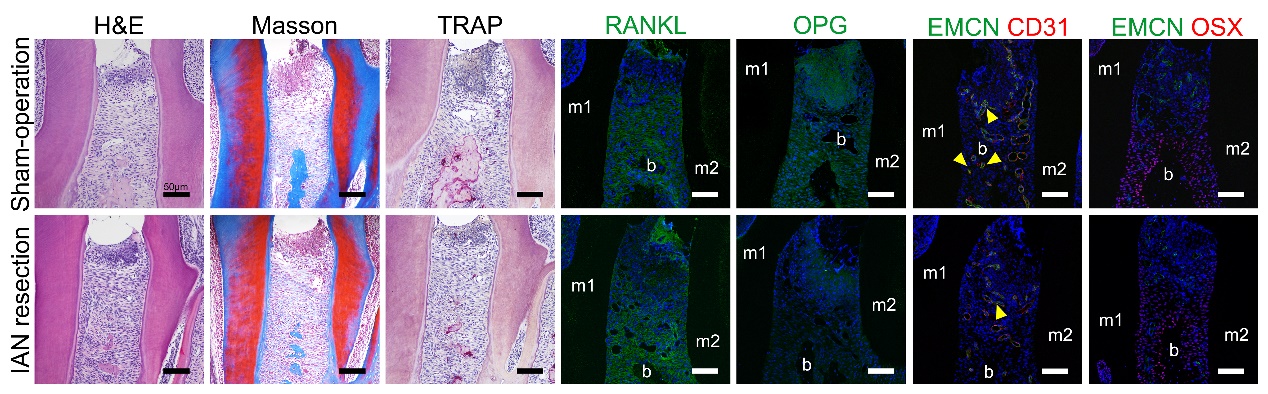


**Figure S14.** H&E staining, Masson's trichrome staining, TRAP staining, and immunofluorescence staining showed aggravation of bone loss, aggregation of osteoclasts, upregulated expression of RANKL, downregulated expression of OPG, reduced density of type H vessels, and fewer OSX-positive osteoprogenitors in the alveolar bone crest on the IAN-denervated side. IAN, inferior alveolar nerve; OPG, osteoprotegerin; RANKL, receptor activators of nuclear factor kappa-B ligand; EMCN, endomucin; OSX, Osterix. b, bone; m1, the first molars; m2, the second molars.


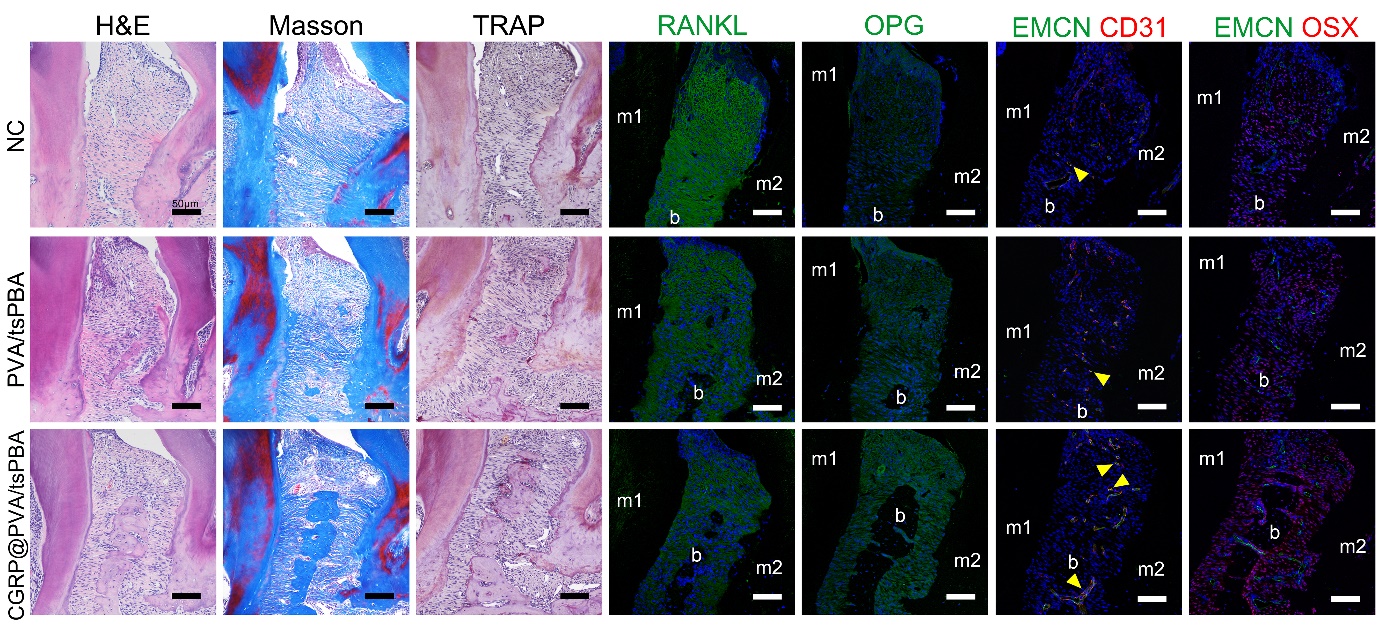


**Figure S15.** H&E staining, Masson's trichrome staining, TRAP staining, and immunofluorescence staining showed enhanced bone regeneration, fewer osteoclasts, reduced expression of RANKL, increased expression of OPG, higher density of type H vessels, and a greater number of OSX-positive osteoprogenitors in the alveolar bone crest of the CGRP@PVA/tsPBA group. PVA, poly (vinyl alcohol); OPG, osteoprotegerin; RANKL, receptor activators of nuclear factor kappa-B ligand; EMCN, endomucin; OSX, Osterix. b, bone; m1, the first molars; m2, the second molars.


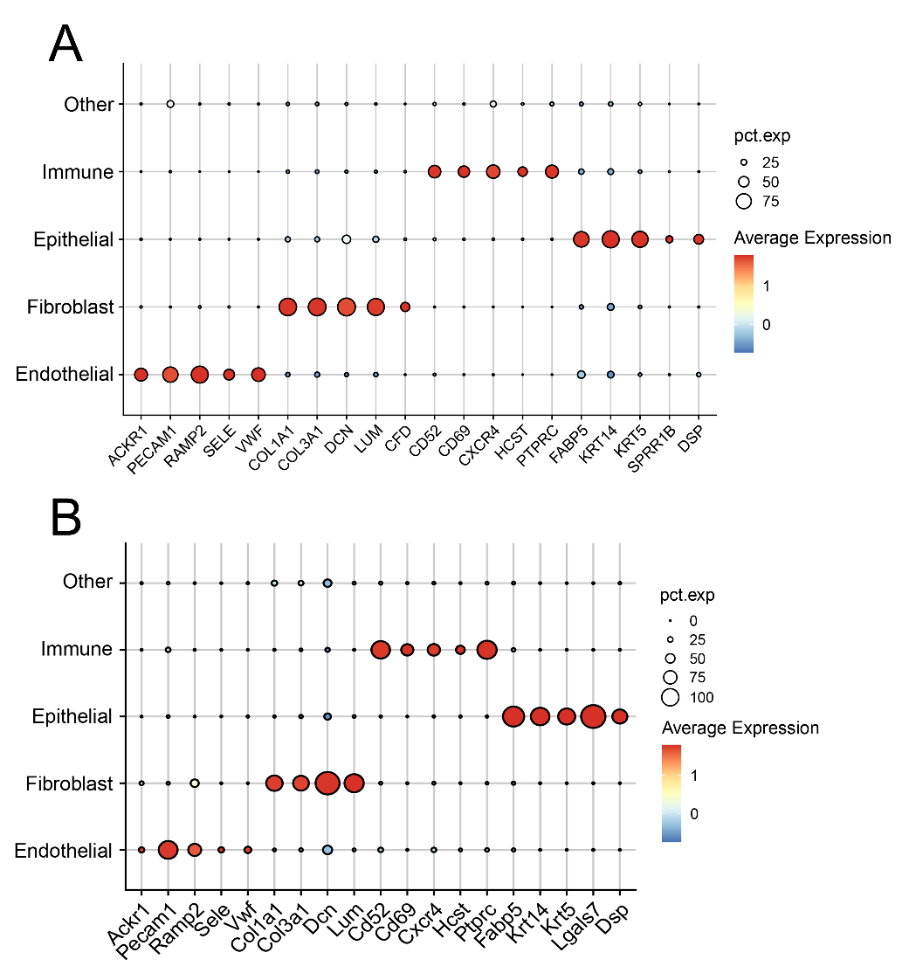


**Figure S16.** Cell type marker gene bubble plot. A) Bubble chart displaying human marker genes. B) Bubble chart of mouse marker genes.


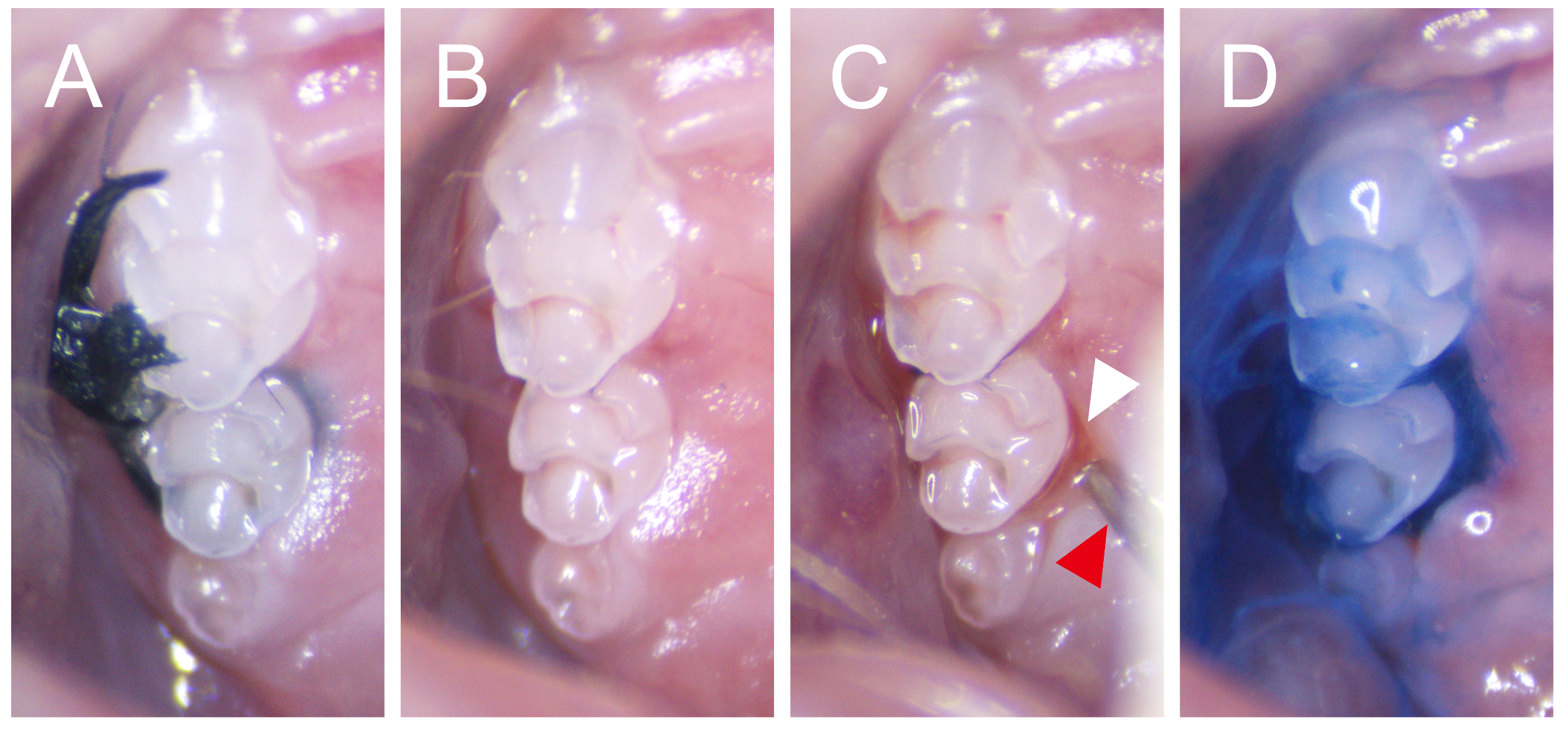


**Figure S17.** Procedures for the injection of PVA/tsPBA hydrogels, stained with blue dye. A) The second molar with the ligature in place. B) Removal of the ligature. C) Exposure of the periodontal pockets. D) A representative image showing the hydrogel remaining in the deep periodontal pockets after injection. White triangle: deep periodontal pockets; Red triangle: needle tip.
